# LYVE1 ectodomain shedding blunts lymphatic transmigration and clearance of macrophages during kidney injury

**DOI:** 10.1172/jci.insight.195176

**Published:** 2026-01-22

**Authors:** Jing Liu, Yuqing Liu, Wenqian Zhou, Saiya Zhu, Jianyong Zhong, Haichun Yang, Annet Kirabo, Valentina Kon, Chen Yu

**Affiliations:** 1 Department of Nephrology, Tongji Hospital, School of Medicine, Tongji University, Putuo District, Shanghai, China.; 2 Department of Pediatrics and; 3 Department of Medicine, Vanderbilt University Medical Center, Nashville, Tennessee, USA.

**Keywords:** Inflammation, Nephrology, Vascular biology, Fibrosis, Lymph, Macrophages

## Abstract

Although renal fibrosis is predominantly driven by the accumulated inflammatory cells that secrete proinflammatory factors within the kidney, the key mechanisms underlying macrophage clearance from the kidney are not well understood. The interaction of hyaluronan with lymphatic endothelial hyaluronan receptor 1 (LYVE1) constitutes a critical initial step in macrophage adhesion and removal by lymphatic vessels. This study investigates alterations in LYVE1 during kidney disease and elucidates its role in macrophage trafficking. Three renal fibrosis models demonstrated a reduction in full-length LYVE1 and an increase in the soluble LYVE1 fragment. Immunostaining of fibrotic kidneys showed significantly reduced expression of soluble LYVE1 compared with the intracellular fragment (Cyto-LYVE1), demonstrating ectodomain shedding of LYVE1 in vivo and in vitro. Functionally, human lymphatic endothelial cells exposed to TGF-β1 exhibited a significant decrease in macrophage adhesion and transendothelial migration compared with controls. Mechanistic analyses identified increased matrix metalloproteinase 9 (MMP9) in renal injury as a key upstream regulator of LYVE1 shedding. MMP9 inhibitors reduced LYVE1 shedding, enhanced macrophage adhesion and trafficking, and mitigated macrophage accumulation and disease progression. In conclusion, MMP9-induced LYVE1 shedding is linked to progressive kidney fibrosis and macrophage accumulation. LYVE1 shedding inhibitors offer potential as therapeutic agents for mitigating immune overload and kidney fibrosis.

## Abstract 2

LYVE1 shedding may block macrophage clearance via lymphatics, explaining the paradox of both increased macrophages and lymphatic growth in kidney fibrosis.

## Graphical abstract

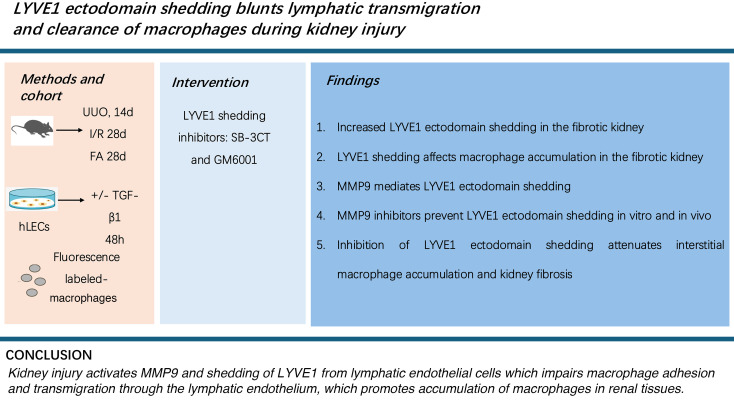

## Introduction

A key factor in the progression of kidney damage is infiltration and accumulation of immune cells. Macrophages, the primary immune cell infiltrating into the kidney parenchyma, are key players that damage glomerular and tubular cells by releasing inflammatory factors including TNF-α and IL-1β in the early stages of kidney damage (1). Macrophages also participate in the chronic phases of fibrosis of diabetic nephropathy (2), IgA nephropathy (3), and post-renal transplantation (4). Consistently, animal models show that reducing macrophage abundance during the fibrotic phase can protect against collagen buildup in different disease models, including ischemia/reperfusion (I/R) (5) and unilateral ureteral obstruction (UUO) (6). Although other cells within the kidneys, including tubular epithelial cells and pericytes, have been postulated to contribute to the myofibroblast pool and thus progressive kidney damage in various disease settings and at different stages of disease, reducing the conversion of macrophages to myofibroblasts remains a potential therapeutic option for fibrosis (7, 8).

The accumulation of macrophages within the kidneys reflects the influx of cells from the bloodstream and local proliferation (9–11). Considerable research has focused on the pathways of macrophage chemotaxis in relation to kidney fibrosis (12–15). In contrast, much less is understood about mechanisms for macrophage clearance. Recently, accumulating studies stress the critical role of the lymphatic system in clearing immune cells, macromolecules, solutes, and fluid from the interstitium of various organs and link lymphatic dysfunction to the development and progression of chronic and fibrotic cardiovascular, hepatic, and intestinal diseases (16–20). Numerous experimental and clinical studies have documented an increase in the renal lymphatic vessel (LV) network following kidney disease (16). Interestingly, interventions that expand the lymphatic network, e.g., exogenous administration of vascular endothelial growth factor C (VEGFC) or activation of VEGF receptor-3 (VEGFR3), reduce inflammation and subsequent fibrosis in UUO injury (21–23). Similarly, VEGFC administration reduced inflammation and cystic formation in *Cys1^cpk/cpk^* mice, a model of autosomal recessive polycystic kidney disease (24). The beneficial effects of these interventions were attributed to the lymphatic removal of inflammatory cells and inflammatory molecules from the kidney interstitium (21). Importantly, homing of immune cells toward the LVs involves the lymphatic growth factor VEGFC, which is a strong activator of adhesion molecules on lymphatic endothelial cells (LECs) (21). Interestingly, our previous research has shown that kidney injuries in rats and mice impair lymphatic pump function that promotes stagnation of immune cells, sodium, water, and lipids within the injured kidneys (25–29). Thus, although it is understood that kidney disease leads to immune cell accumulation and lymphatic vascular growth that supports clearance of immune cells, we sought to understand possible impairment in the lympho-macrophage trafficking processes that occur during kidney disease.

Lymphatic endothelial hyaluronan receptor 1 (LYVE1) is a transmembrane protein that binds hyaluronan (HA) expressed by macrophages and promotes macrophage adhesion to LVs. Normally, macrophage HA glycocalyx binds the LYVE1 HA-binding motif that aids their adhesion and entry into the LVs (30). Deletion of LYVE1 impedes inflammatory cell entry into the lymphatic capillaries and has been observed to worsen inflammation and cardiac dysfunction in myocardial infarction models (31). The LYVE1 protein contains an HA motif in its N-terminal sequence, while its cytoplasmic domain is located in the C-terminal polypeptide sequence (32). Shedding and loss of the HA-binding sites of LYVE1 have been documented in psoriasis as well as in corneal diseases (33, 34). Moreover, leukocyte shedding of CD44 adhesion molecule, which has 41% structure similarity to LYVE1 (32), is widely reported and has been linked to abnormal cell migration, intense inflammation, and tumor progression (35, 36). Whether LYVE1 shedding occurs in kidney injury is unknown. Interestingly, however, we recently reported elevated levels of serum soluble LYVE1 in patients with moderate to severe kidney fibrosis compared with those with only mild fibrosis or no fibrosis, suggesting an association between soluble LYVE1 and progressive fibrosis in the kidney (37). Here, we hypothesized that shedding of the LYVE1 ectodomain from LECs impairs LYVE1/HA–mediated macrophage adhesion, transport, and clearance, which serves to exacerbate kidney injury and progressive fibrosis.

## Results

### Increased LYVE1 ectodomain shedding in the fibrotic kidney.

**Figure 1:**
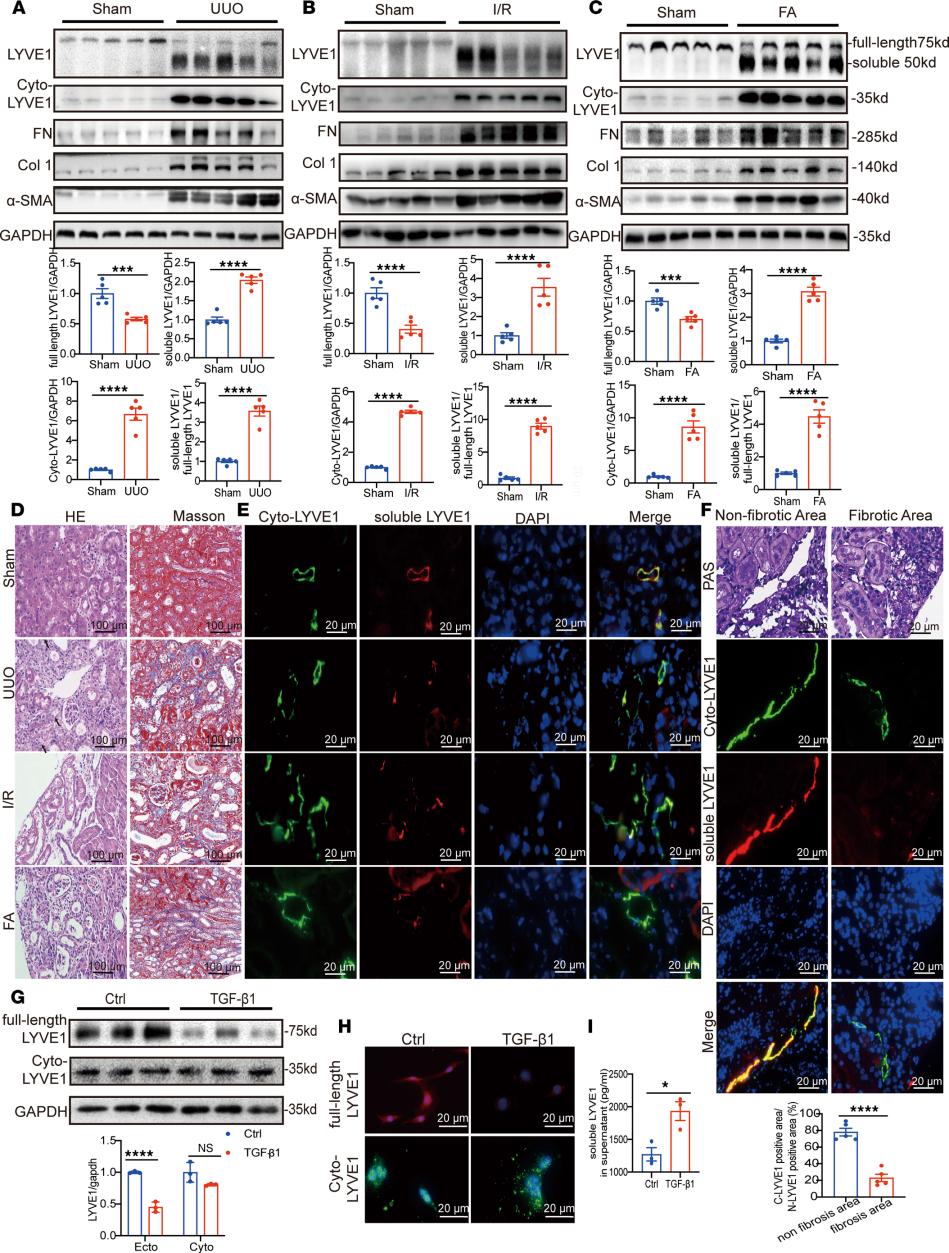
Lymphatic vessel LYVE1 shedding in three models of kidney fibrosis. (**A**–**C**) Western blots of full-length LYVE1, cleaved soluble LYVE1, and Cyto-LYVE1 as well as fibrotic markers FN, Col I, and α-SMA in sham versus UUO mice (**A**), sham versus I/R (**B**), and sham versus FA (**C**). (**D**) H&E and Masson’s staining showing interstitial fibrosis with the 3 injury models (arrows) (original magnification, ×200; scale bars: 100 μm). (**E**) Costaining of Cyto- (red) and soluble LYVE1 (green) in fibrosis models (original magnification, ×1,000; scale bars: 20 μm). (**F**) Periodic acid–Schiff (PAS) staining and immunostaining of Cyto- and soluble LYVE1 in non-fibrotic (left) and fibrotic (right) areas of the same FA-injured kidney (original magnification, ×400; scale bars: 20 μm) and bar graph (*n* = 6). (**G** and **H**) Western blots (**G**) and immunocytochemistry (ICC) (**H**; original magnification, ×1,000; scale bars: 20 μm) of full-length and Cyto-LYVE1 protein expression in hLECs exposed to TGF-β1. (**I**) Soluble LYVE1 in the supernatant of hLECs exposed to TGF-β1 measured by ELISA. *n* = 5 per group for animal experiments and *n* = 3 per group for cell experiments; statistics used included a 2-tailed *t* test. *P < 0.05, **P < 0.01, ***P < 0.001, ****P < 0.0001.

**Figure 2:**
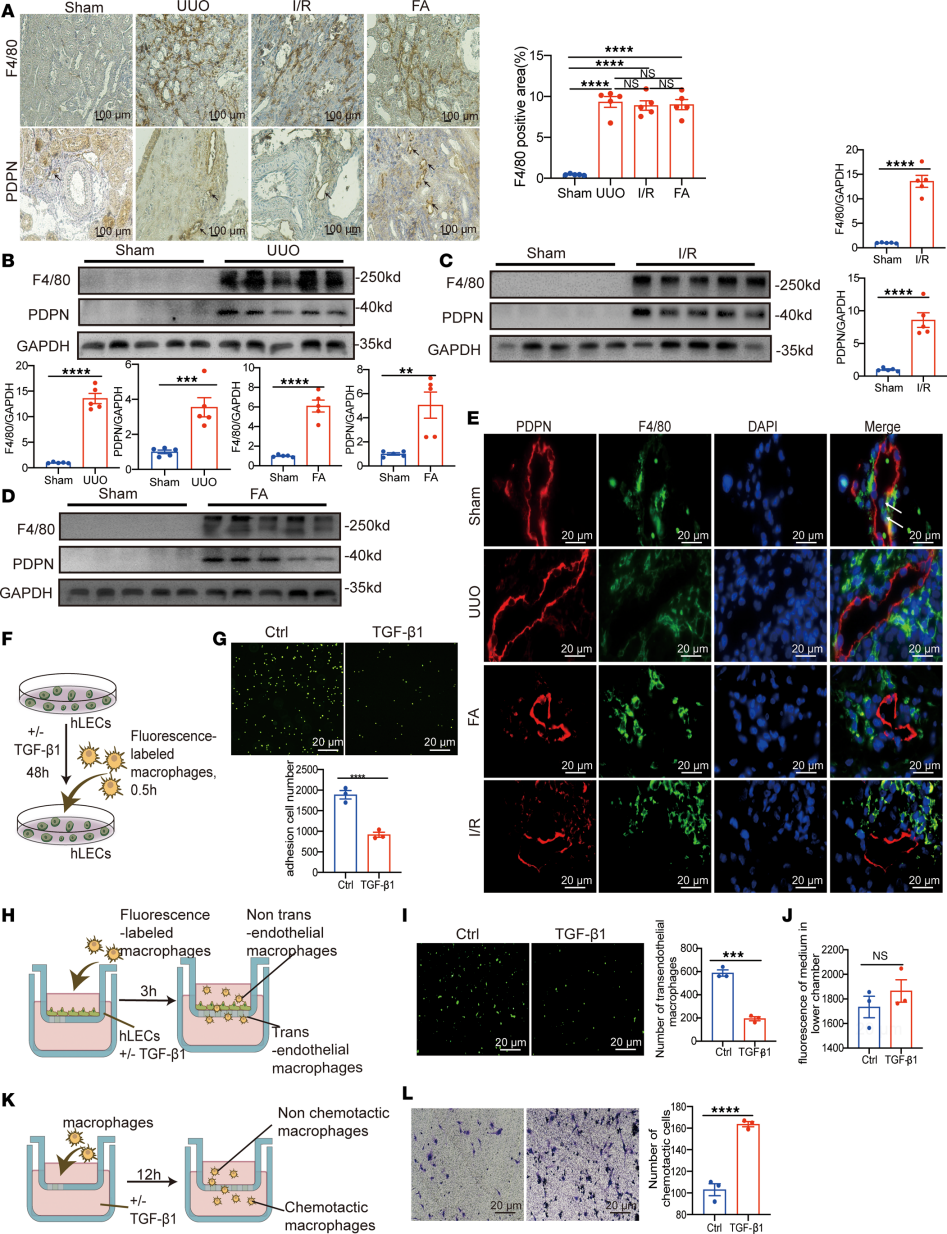
Impaired macrophage transendothelial trafficking in the fibrotic kidney. (**A**) Kidney fibrosis increases macrophage accumulation and lymphangiogenesis (PDPN staining, black arrows) in 3 kidney injury models (original magnification, ×400; scale bars: 100 μm). (**B**–**D**) Western blotting confirms increased F4/80 and PDPN. (**E**) Colocalization of PDPN (red) and F4/80 (green) by immunohistochemical staining. F4/80^+^ macrophages appear in lymphatic lumen (white arrows) of sham mice but not in fibrotic kidneys, which rather show prominent F4/80^+^ aggregation surrounding LVs. Scale bars: 20 μm. (**F**) Schematic illustrating macrophage and LEC adhesion experiment. (**G**) Macrophage adhesion to hLECs with or without TGF-β1 shows reduced number of macrophages adhering to hLECs. Scale bars: 20 μm. (**H**) Schematic diagram illustrating trans-lymphatic endothelial cell migration by macrophages. (**I**) Trans-endothelium experiments of macrophage adhesion to hLECs with or without TGF-β1 show a reduced number of migrated macrophages. Scale bars: 20 μm. (**J**) Fluorescence reading of medium in the lower chamber is similar in two groups. (**K**) Schematic diagram illustrating macrophage chemotaxis assay. (**L**) Under the chemotactic effect of TGF-β1, the number of macrophages migrating to the lower chamber of the Transwell increased significantly. Scale bars: 20 μm. *n* = 5 per group for animal experiments and *n* = 3 per group for cell experiments; statistics used included a 2-tailed *t* test (2 groups, in **B**–**D**, **G**, **I**, **J**, and **L**) or 1-way ANOVA (multiple groups, in **A**). *P < 0.05, **P < 0.01, ***P < 0.001, ****P < 0.0001.

In view of our findings that patients with kidney fibrosis have elevated levels of serum soluble LYVE1 (37), we undertook studies to investigate the effects of kidney injury on LYVE1 ectodomain shedding in 3 distinct mouse models of kidney fibrosis. The UUO model demonstrates rapid progression of tubular atrophy, matrix deposition, and fibrosis that mirrors the sequence of fibrotic events seen in humans (38, 39). The I/R model illustrates the transition from acute to chronic kidney injury and includes the key features of interstitial fibrosis (40). The patchy interstitial fibrosis that characterizes folic acid–induced (FA-induced) nephropathy is used to simultaneously assess the fibrotic and non-fibrotic regions within the same kidney (41, 42). This provides a versatile framework to examine chronic kidney diseases (40, 43). Each model exhibited prominent fibrotic characteristics with increased expression of fibronectin (FN), collagen I (Col I), and α-smooth muscle actin (α-SMA), and interstitial fibrosis (Figure 1, A–D, and Supplemental Figure 1, A–C; supplemental material available online with this article; https://doi.org/10.1172/jci.insight.195176DS1). We used two LYVE1 antibodies, one targeting the intracellular fragment, Cyto-LYVE1, and the other targeting the extracellular fragment, including both full-length (75 kDa) and cleaved soluble fragment (50 kDa). Each of the fibrosis models showed a significant increase in the Cyto-LYVE1 fragment (Figure 1, A–C), consistent with LV expansion extensively documented following kidney injury and disease (16, 21, 44–46). LYVE1 Western blot also showed decreased full-length LYVE1 and increased cleaved soluble fragment in each of the fibrosis models (Figure 1, A–C). Immunofluorescent staining of uninjured kidneys of normal mice consistently demonstrated colocalization of soluble LYVE1 and Cyto-LYVE1. In contrast, the fibrotic kidneys had significantly reduced expression of soluble LYVE1 compared with Cyto-LYVE1 (Figure 1E). The fibrotic and non-fibrotic regions were characterized by periodic acid–Schiff staining, and increased LYVE1 shedding was observed in fibrotic regions compared with non-fibrotic regions of the same kidney in the FA injury model (Figure 1F). Western blot analysis of total kidney lysates demonstrated increased cytoplasmic LYVE1 in fibrotic kidneys, findings that are in line with the increased number of LVs as confirmed by podoplanin (PDPN) Western blot and immunostaining in whole kidneys (Figure 2, A–D). In contrast, immunofluorescence images shown in Figure 1E were designed to compare LYVE1 localization at the single-vessel level within a distinct microenvironment. These images reveal that LVs located in fibrotic regions displayed a clear loss of ectodomain LYVE1 staining despite preserved cytoplasmic LYVE1 signal. This localized pattern suggests that ectodomain LYVE1 is preferentially shed from LVs within fibrotic areas, while total cytoplasmic LYVE1 levels remain elevated at the whole-kidney level reflecting increased LV density in the diseased kidney.

It is well known that TGF-β1 is elevated after kidney injury (47). To more directly assess LYVE1 hydrolysis, we measured LYVE1 shedding in cultured human LECs (hLECs) exposed to TGF-β1. Complementing the in vivo results, TGF-β1 exposure decreased LEC expression of full-length LYVE1, whereas Cyto-LYVE1 levels remained unchanged. These gene expression studies were confirmed by Western blotting and immunocytochemistry, reinforcing the decreased LEC expression of full-length LYVE1 and unchanged Cyto-LYVE1 levels (Figure 1, G and H). Soluble LYVE1 was significantly increased in the conditioned medium of TGF-β1–exposed LECs (Figure 1I). Together, these findings demonstrate that LYVE1 ectodomain shedding occurs in fibrotic kidneys in vivo and in TGF-β1–stimulated cultured LECs.

### LYVE1 shedding affects macrophage accumulation in the fibrotic kidney.

LYVE1 is crucial for macrophage docking, entry, and removal by lymphatics that prevent macrophage accumulation (31). Depletion or compromise in LYVE1 integrity leads to pathophysiological consequences (33, 34). As expected, in our study, UUO, I/R, and FA all showed significant macrophage accumulation as assessed by immunohistochemical staining and F4/80 Western blot (Figure 2, A–D). Also, LV number was significantly increased in fibrotic kidneys, as indicated by the expression of the LV marker PDPN (Figure 2, A–D). Interestingly, F4/80^+^ macrophages were conspicuous not only around PDPN^+^ LVs but also within the lymphatic lumen of sham mice. In contrast, although the total number of macrophages was significantly increased in fibrotic kidneys, their localization was primarily outside the LV with only occasional cells observed within the vessel lumen (Figure 2E).

To better understand the pathways of lymphatic transport of macrophages, we examined macrophage attachment and migration with LECs. Cultured hLECs with or without TGF-β1 were cocultured with differentiated THP-1 macrophages. Adherent macrophages (visualized with green dye) were significantly reduced in hLECs exposed to TGF-β1 compared with control cells (Figure 2, F and G) in a time-dependent manner (Supplemental Figure 1D). Furthermore, transendothelial migration assays revealed that significantly fewer macrophages traversed TGF-β1–exposed hLECs compared with control hLEC layers (Figure 2, H and I). Notably, the fluorescence intensity in the medium (as opposed to the underside of the Transwell chamber) was not different between the two groups, indicating that the reduced number of traversed cells quantitated in the migration assay was not because of fewer cells drifting to the bottom of the chamber (Figure 2J). More importantly, TGF-β1 significantly enhanced macrophage chemotaxis toward the conditioned medium, indicating a strong chemoattractant effect independent of LYVE1 shedding (Figure 2, K and L). Together with data showing LYVE1 hydrolysis (Figure 1), these findings provide a coherent mechanistic explanation for our in vivo observation of increased macrophage accumulation around LVs in fibrosis models (Figure 2E), supporting the concept that TGF-β1 drives macrophage attraction but impairs their lymphatic clearance.

### MMP9 mediates LYVE1 ectodomain shedding.

**Figure 3:**
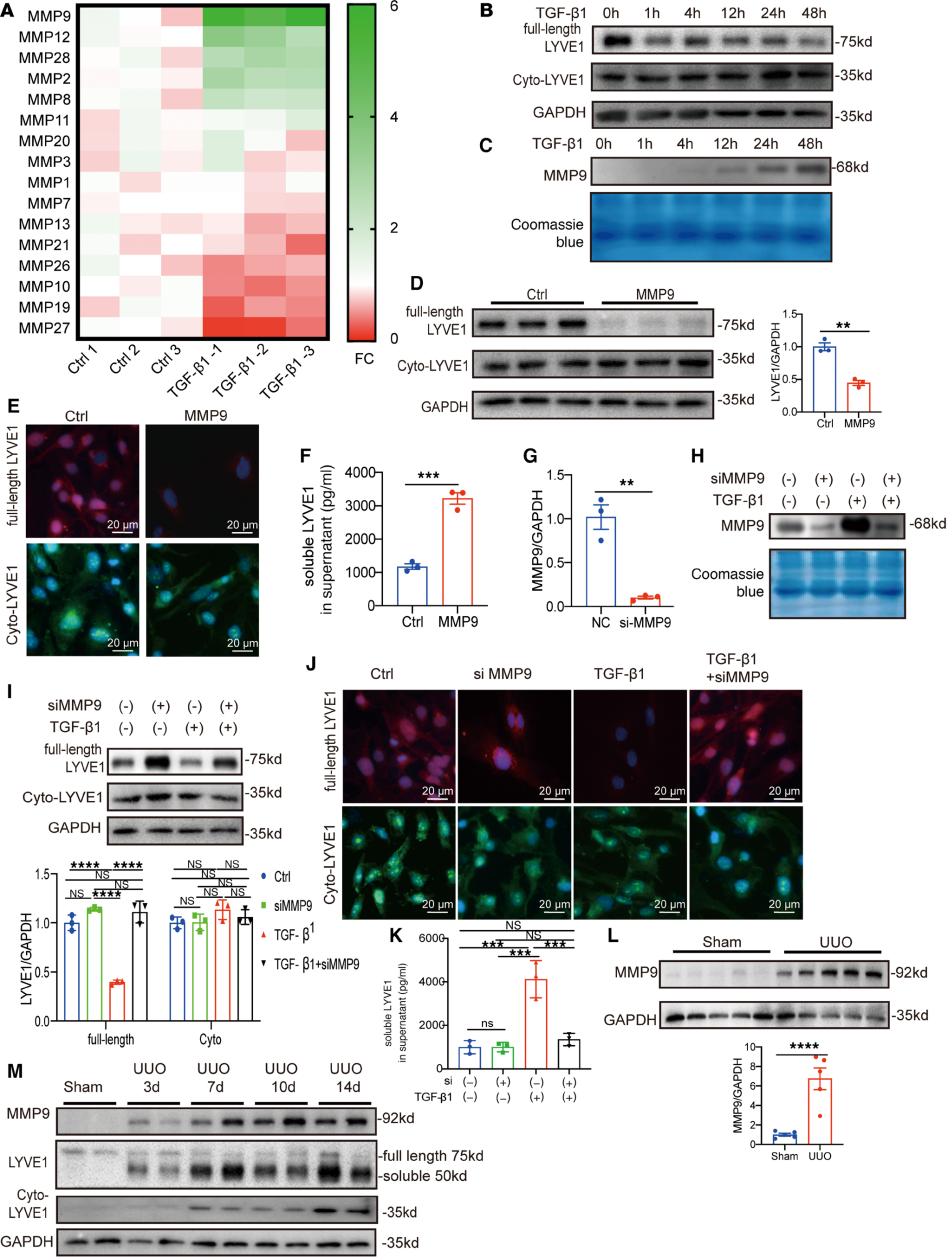
MMP9 mediates LYVE1 ectodomain shedding. (**A**) Fold change of RNA expression of MMPs on LECs after TGF-β1 stimulation. (**B**) Time course for full-length LYVE1 and Cyto-LYVE1 in TGF-β1–treated hLECs assessed by Western blot. (**C**) MMP9 in supernatant of TGF-β1–treated hLECs measured by gelatin zymography. (**D**) Full-length and Cyto-LYVE1 protein expression of MMP9-exposed hLECs assessed by Western blot. (**E**) Full-length and Cyto-LYVE1 protein expression of MMP9-treated hLECs assessed by ICC (original magnification, ×1,000; scale bars: 20 μm). (**F**) Soluble LYVE1 in supernatant of MMP9-exposed hLECs assessed by ELISA. (**G**) MMP9 expression in hLECs after siRNA-mediated knockdown. (**H**) MMP9 in the supernatant of hLECs treated with siMMP9 assessed by gelatin zymography. (**I**) Full-length and Cyto-LYVE1 protein expression of hLECs treated with siMMP9 inhibitors assessed by Western blot. (**J**) Full-length and Cyto-LYVE1 protein expression of hLECs treated with siMMP9 assessed by ICC (original magnification, ×1,000; scale bars: 20 μm). (**K**) Soluble LYVE1 in the supernatant of hLECs treated with siMMP9 measured by ELISA. (**L** and **M**) Western blot of MMP9, LYVE1, and Cyto-LYVE1 in UUO at the end of the experiment (14 days) (**L**) and over the course of the study (**M**). *n* = 3 per group for cell experiments; statistics used included a 2-tailed *t* test (2 groups, in **D**, **F**, **G**, and **L**) or 1-way ANOVA (multiple groups, in **I** and **K**).*P < 0.05, **P < 0.01, ***P < 0.001, ****P < 0.0001.

Given the critical role of LYVE1 ectodomain shedding in macrophage transport, we aimed to identify the upstream regulators of this process. The matrix metalloproteinase (MMP) family is known to cleave transmembrane proteins and release their soluble forms in physiological and pathological conditions (48). To determine which MMP is preferentially induced in our model, we performed quantitative PCR analysis of LECs for MMP family members previously reported to have sheddase activity (including MMP9, MMP12, MMP28, MMP2, MMP8, MMP11, MMP20, MMP3, MMP1, MMP7, MMP13, MMP21, MMP26, MMP10, MMP19, and MMP27) (49) following TGF-β1 stimulation. Among these, MMP9 showed the greatest induction (Figure 3A). Moreover, MMP9 regulates the shedding of the leukocyte HA receptor homologous to LYVE1, CD44 (50). We focused on MMP9, which has been linked to kidney fibrosis and is induced by TGF-β1 (51–53). In vitro, gelatinase assays demonstrated a time-dependent increase in MMP9 levels in hLECs exposed to TGF-β1, findings that correlated with the increase in LYVE1 shedding (Figure 3, B and C). To assess whether MMP9 regulates LYVE1 shedding, we supplemented the culture medium with activated MMP9. MMP9 caused a marked reduction of full-length LYVE1 in the cell lysates (Figure 3, D and E) and increased free soluble LYVE1 in the supernatant (Figure 3F). siRNA-mediated knockdown of MMP9 in hLECs resulted in approximately 90% reduction of MMP9 mRNA expression (quantitative PCR shown in Figure 3G) and a marked decrease in protein levels (Western blot shown in Figure 3H). These results confirm efficient silencing of MMP9. Furthermore, transfection of hLECs with MMP9 siRNA significantly reduced LYVE1 ectodomain shedding (Figure 3, I–K).

In vivo, MMP9 expression was significantly elevated in whole protein lysates extracted from UUO kidneys compared with normal kidneys (Figure 3L), and the expression increased as the disease progressed (Figure 3M). Together, these studies support the idea that MMP9 within kidneys mediates the shedding of LYVE1 ectodomain during fibrosis.

### MMP9 inhibitors prevent LYVE1 ectodomain shedding in vitro and in vivo.

**Figure 4:**
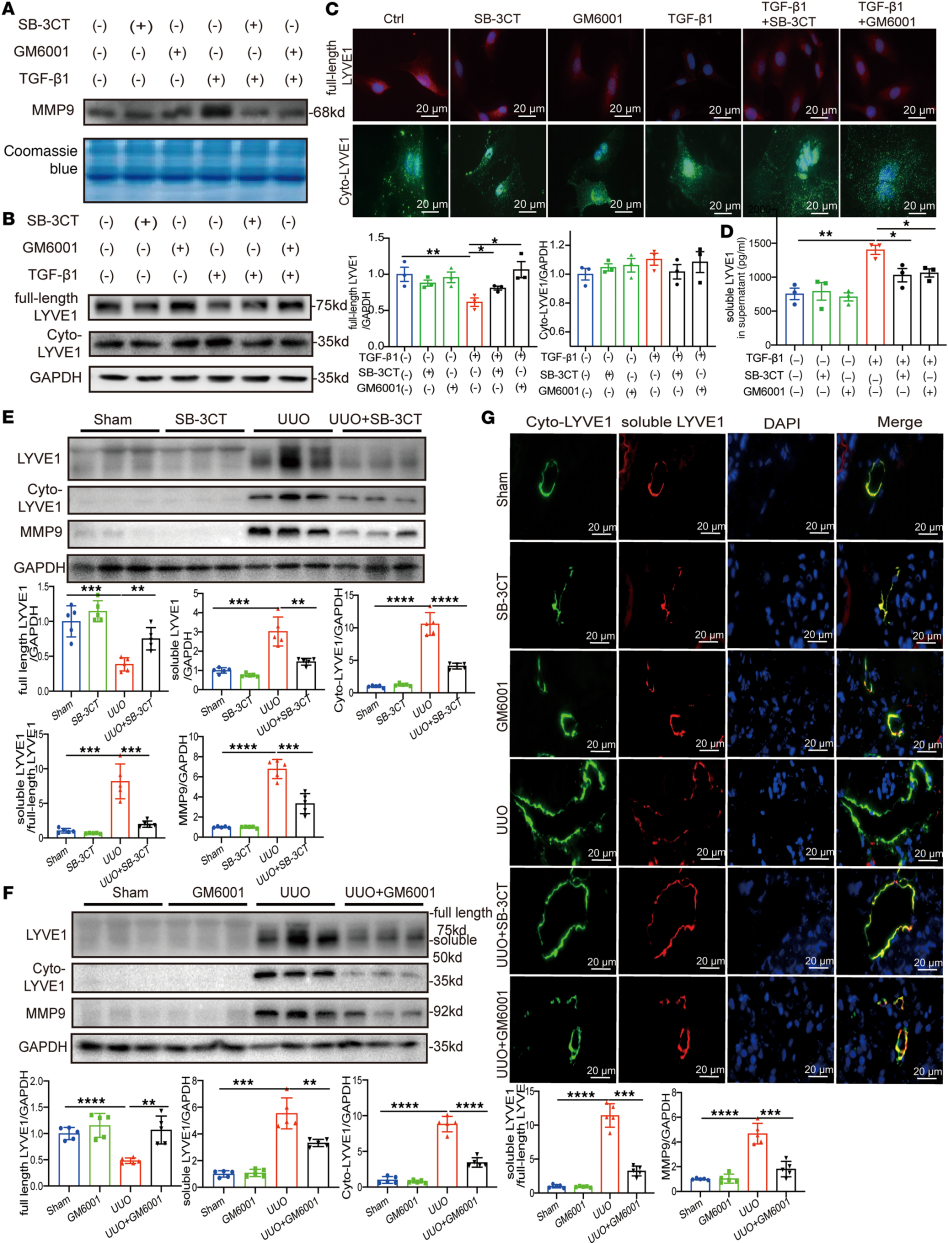
MMP9 inhibition prevents LYVE1 ectodomain shedding in vivo and in vitro. (**A**) Gelatin zymography measured MMP9 in the supernatant of hLECs treated with the MMP9 inhibitors SB-3CT and GM6001. (**B**) Western blot measured full-length and Cyto-LYVE1 protein expression of hLECs treated with the MMP9 inhibitors SB-3CT and GM6001. (**C**) ICC (original magnification, ×1,000; scale bars: 20 μm) measured full-length and Cyto-LYVE1 protein expression of hLECs treated with SB-3CT and GM6001. (**D**) ELISA measured soluble LYVE1 in the supernatant of hLECs treated with SB-3CT and GM6001. (**E** and **F**) Western blot of full-length and cleaved soluble LYVE1 and Cyto-LYVE1 in the sham group, UUO group, UUO with the specific shedding inhibitor SB-3CT group (**E**), and UUO with the nonspecific shedding inhibitor GM6001 group (**F**). (**G**) Cyto- and soluble LYVE1 costaining showed reduced LYVE1 shedding by SB-3CT or GM6001 (original magnification, ×1,000; scale bars: 20 μm). *n* = 5 per group for animal experiments and *n* = 3 per group for cell experiments; statistics used included 1-way ANOVA. *P < 0.05, **P < 0.01, ***P < 0.001, ****P < 0.0001.

Given that MMP9 appears to be a key protease mediating LYVE1 shedding in kidney fibrosis, we investigated whether MMP9 inhibitors could attenuate LYVE1 hydrolysis. We used the specific MMP9 inhibitor SB-3CT and the nonspecific inhibitor GM6001 (which has inhibition function for most MMPs) in our study. In vitro, both SB-3CT and GM6001 decreased TGF-β1–induced MMP9 secretion by LECs into the culture medium (Figure 4A). They both also decreased TGF-β1–induced LYVE1 ectodomain shedding in hLECs (Figure 4, B and C) and lowered the level of soluble LYVE1 in the supernatant (Figure 4D). Among the 3 models, the UUO model exhibits uniform kidney damage and is marked by progressive interstitial inflammation and fibrosis (38, 54). Because of these characteristics, we selected the UUO model for further rescue experiments. UUO mice were treated with SB-3CT and GM6001 for a duration of 14 days (55, 56).

In vivo, the inhibitors significantly reduced expression of cleaved soluble LYVE1 (50 kDa) in Western blot (Figure 4, E and F), preserved integrity of soluble LYVE1 immunostaining, and enhanced colocalization of full-length LYVE1 and cellular LYVE1 (Figure 4G). These data support the idea that the MMP9 inhibitor reduced LYVE1 ectodomain shedding.

### Inhibition of LYVE1 ectodomain shedding attenuates interstitial macrophage accumulation and kidney fibrosis.

**Figure 5:**
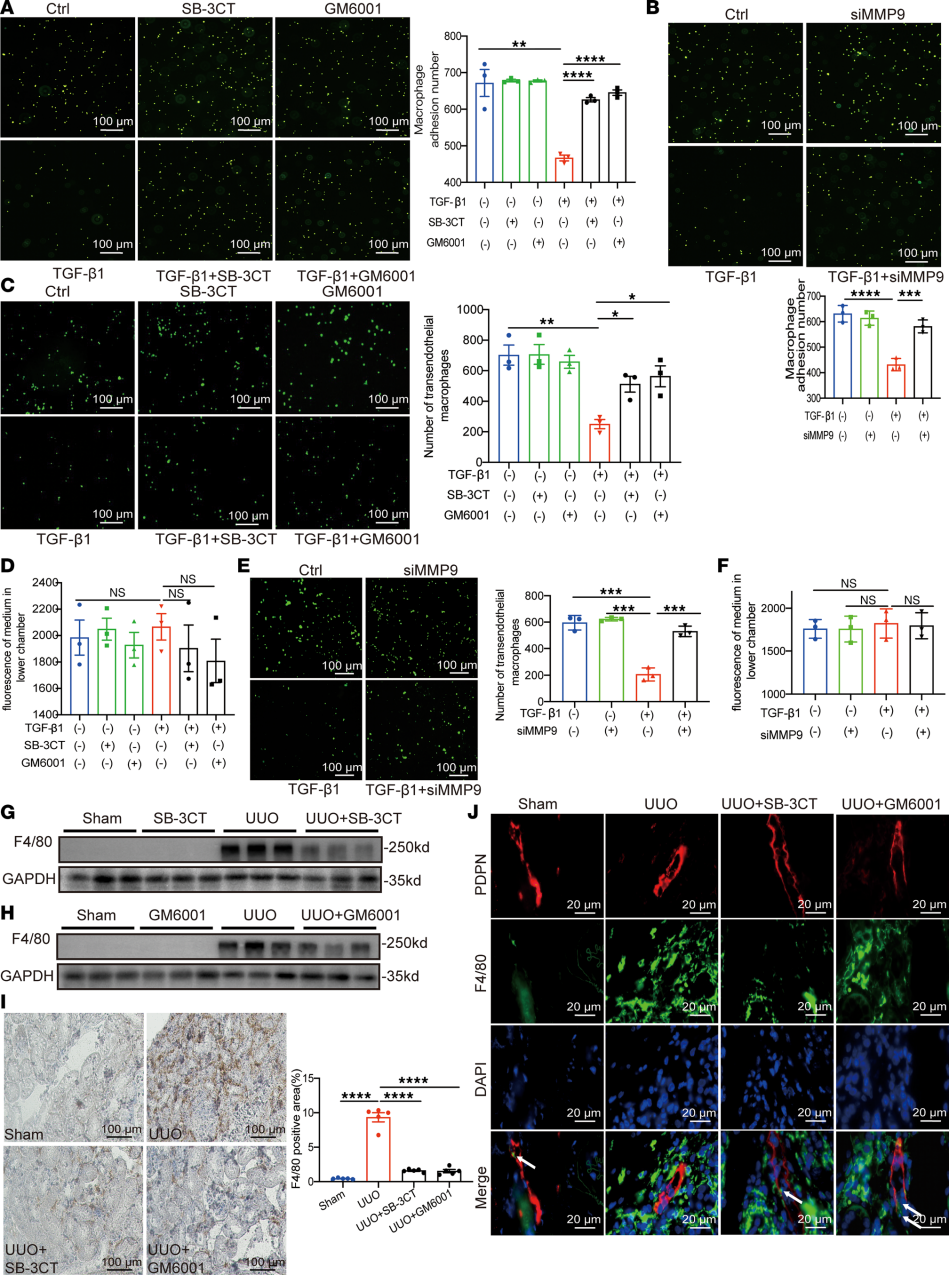
Macrophage accumulation is ameliorated by shedding inhibitors in vivo. F4/80^+^ macrophage aggregation was more prominent around the LVs in UUO mice compared with the other groups. (**A**) Left: In vitro adhesion experiments of macrophages and hLECs with TGF-β1 and SB-3CT or GM6001. Right: Bar graph of adhesion experiments; each point represents the average adhesion number of 5 random visual fields per well, assessed in 3 wells in each group. Scale bars: 100 μm. (**B**) Experiments of adhesion of macrophages to hLECs with TGF-β1 and siMMP9 treatment. Scale bars: 100 μm. (**C** and **E**) Trans-endothelium experiments of macrophage adhesion to hLECs with or without SB-3CT or GM6001 (**C**) and TGF-β1 and siMMP9 (**E**). Scale bars: 100 μm. (**D** and **F**) Fluorescence reading of culture medium in the lower chamber of trans-endothelium experiments of macrophage adhesion to hLECs with or without SB-3CT or GM6001 (**D**) and TGF-β1 and siMMP9 (**F**). (**G** and **H**) F4/80 protein expression of sham, UUO, UUO plus SB-3CT (**G**), or UUO plus GM6001 (**H**). (**I**) Macrophage marker F4/80 staining in kidneys of sham, UUO, UUO plus SB-3CT, or UUO plus GM6001. Scale bars: 100 μm. (**J**) F4/80^+^ macrophages (green) were observed in the lymphatic lumen (PDPN, red) (white arrows) of sham mice and mice treated with UUO plus SB-3CT or UUO plus GM6001 but not in untreated UUO mice (original magnification, ×1,000; scale bars: 20 μm). *n* = 5 per group for animal experiments; statistics used included 1-way ANOVA. *P < 0.05, **P < 0.01, ***P < 0.001, ****P < 0.0001.

In complementary in vitro studies, exogenous MMP9 significantly reduced macrophage adhesion to hLECs (Supplemental Figure 1E). In contrast, inhibition (Figure 5A) or knockdown of MMP9 with siRNA (Figure 5B) reestablished TGF-β1 impairment of LEC-macrophage adhesion. Both shedding inhibitors and siRNA treatment restored macrophage transendothelial transport reduced by TGF-β1 (Figure 5, C and E). There was no significant difference in the green fluorescence intensity of the lower chamber across the different groups (Figure 5, D and F), indicating that the number of macrophages migrated into the lower chamber was similar.

**Figure 6:**
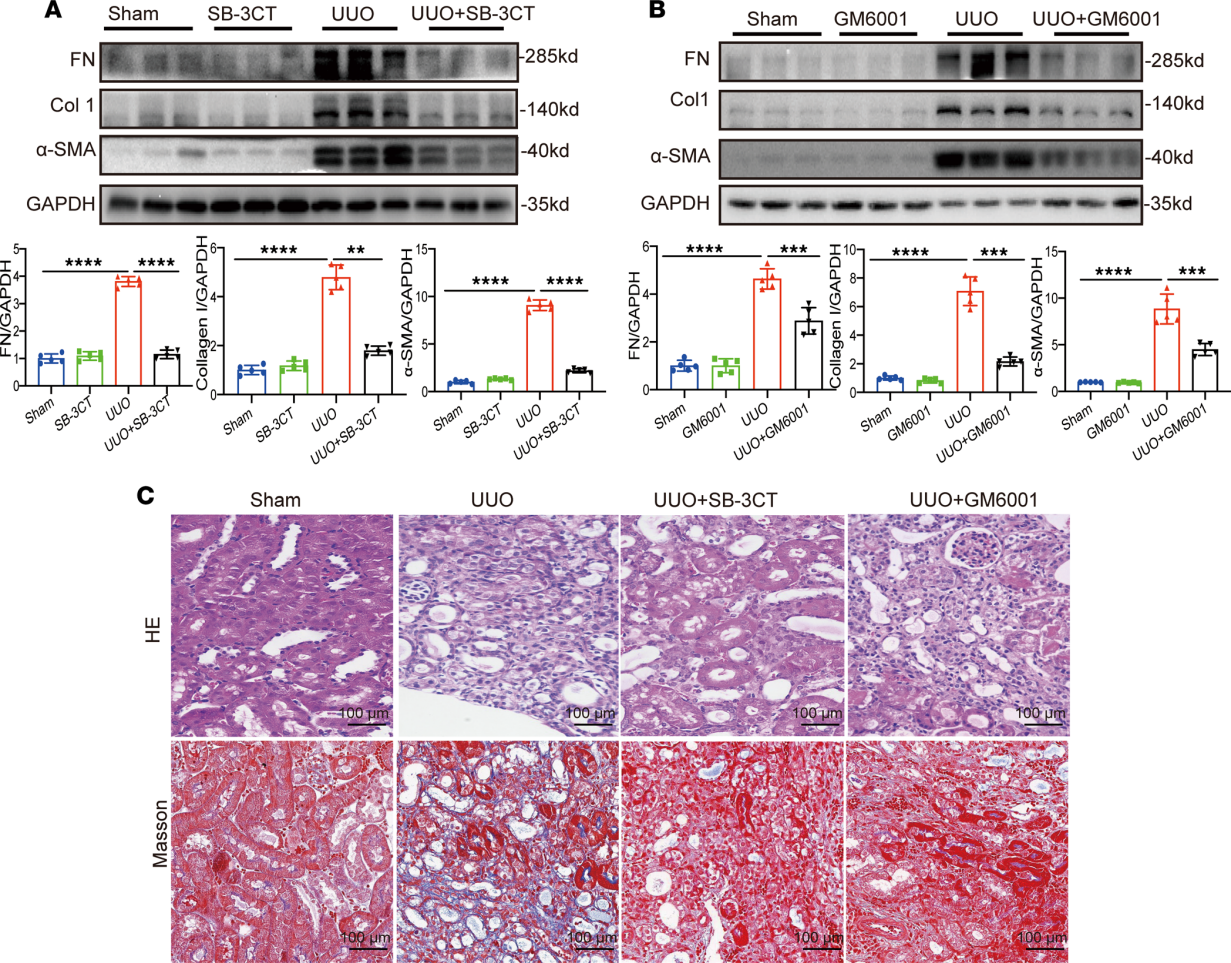
LYVE1 ectodomain shedding inhibition ameliorates renal fibrosis in vivo. (**A** and **B**) Western blot of FN, Col I, and α-SMA in sham group, UUO group, and UUO plus SB-3CT or GM6001 groups. (**C**) H&E and Masson’s staining shows morphological changes in UUO models and UUO plus SB-3CT or GM6001 (original magnification, ×200; scale bars: 100 μm). *n* = 5 per group for animal experiments; statistics used included 1-way ANOVA. *P < 0.05, **P < 0.01, ***P < 0.001, ****P < 0.0001.

In vivo, treatment with the shedding inhibitors, SB-3CT and GM6001, significantly reduced F4/80^+^ macrophage aggregation in UUO-injured mice assessed by immunohistochemistry and Western blot (Figure 5, G–I, and Supplemental Figure 1, F and G). To evaluate whether shedding inhibitors could restore renal lymphatic transport function, we performed PDPN and F4/80 costaining. Shedding inhibitors reduced F4/80^+^ macrophages and lessened the accumulation of macrophages surrounding the renal LVs (Figure 5J). Both inhibitors also reduced the expression of fibrosis-related proteins (FN, Col I, and α-SMA) (Figure 6, A and B) and attenuated tissue damage as shown by H&E and Masson’s staining (Figure 6C). Cumulatively, these results suggest that the inhibitors of LYVE1 ectodomain shedding are effective in alleviating macrophage efflux obstruction and kidney fibrosis.

## Discussion

**Figure 7:**
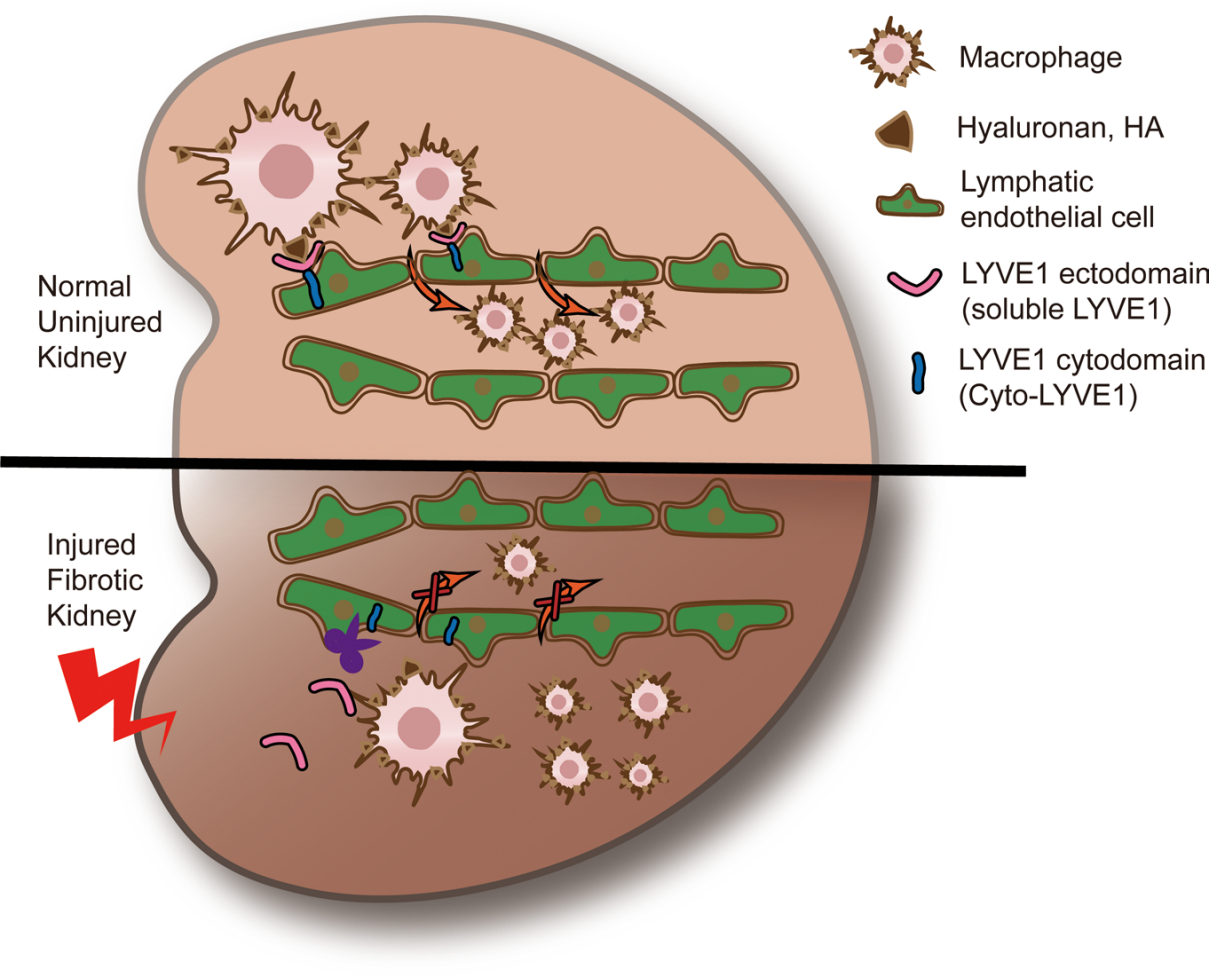
A proposed mechanism for kidney injury–driven macrophage accumulation includes impaired macrophage clearance. Kidney injury activates MMP9 and shedding of LYVE1 from lymphatic endothelial cells, which impairs macrophage adhesion and transmigration through the lymphatic endothelium, which promotes accumulation of macrophages in renal tissues.

The lymphatic system plays a crucial role in immune regulation by enabling the migration of immune cells from tissues to the draining lymph nodes, where antigen-specific immune responses are initiated and maintained. This network also facilitates the clearance of phagocytes, apoptotic cells, and tissue debris, which are essential in the repair of damaged and inflamed tissues. Notably, the expansion of the lymphatic vascular network in patients and animal models of progressive kidney injury is thought to provide a highly efficacious outflow for immune cells (44, 45, 57). Simultaneously, increased macrophage abundance has been linked to progressive fibrosis (7, 12). This apparent paradox prompted us to investigate a potential dysfunction in immune cell clearance by the lymphatic vessels (LVs). Using 3 distinct renal fibrosis models, we provide compelling evidence for LYVE1 shedding from the renal lymphatic endothelial cells, a previously unreported phenomenon. Also, costaining studies showing the separation of LYVE1’s intracellular and extracellular domain fragments (Figure 1E). Complementing these results, Western blot analysis showed a marked reduction in the 75 kDa full-length LYVE1 and a corresponding increase in the 50 kDa soluble LYVE1 fragment (Figure 1, A–C). Importantly, although an increase in the 35 kDa Cyto-LYVE1 fragment, indicating lymphangiogenesis, was documented, this finding also corroborated that the reduction in full-length LYVE1 is not due to a decrease in LV numbers. While LYVE1 shedding has been documented in other circumstances, such as in models of skin lymphangiogenesis and human lymphoproliferative diseases (33), these findings, we believe, are novel in the context of kidney disease (Figure 7).

LYVE1 cleavage appears to be regulated by MMP9, and modulation of MMP9 reduces LYVE1 shedding. Studies have reported that LYVE1 shedding by lymphatic endothelial cells is catalyzed by MT1-MMP (34) and a disintegrin and a metalloproteinase 17 (ADAM17) (33). Moreover, cleavage of membrane proteins, such as CD44, has been linked to ADAMs and MMPs in tissue remodeling, inflammatory responses, and proliferative signaling pathways during organogenesis (58, 59). In another pathophysiological setting, Wong et al. (34) showed that in mouse corneal LVs, LYVE1 is a direct substrate of MT1-MMP, with MT1-MMP–mediated cleavage regulating physiological lymphangiogenesis independently of VEGFR3 signaling. Our investigation revealed no significant increase in MT1-MMP or ADAM17 expression in hLECs exposed to TGF-β1. This led us to explore the role of MMP9, which has been strongly linked to kidney fibrosis (60, 61). Dysregulated MMP9 has been established in acute and chronic renal injury (62). During the later stages of I/R (e.g., days 14, 28, and 56 after I/R), Mmp9-knockout mice exhibited attenuated loss of renal microvessels (62). In addition, MMP2/9 inhibitors or MMP9-neutralizing antibodies significantly reduced macrophage infiltration, tubular epithelial-mesenchymal transition, and renal fibrosis in a UUO model (63). These findings complement our results that MMP9 alleviates kidney fibrosis. We propose a mechanism wherein MMP9 functions as a protease on LVs, shedding LYVE1 to reduce macrophage migration. Our in vitro data demonstrate that TGF-β–induced endogenous MMP9 or exogenous recombinant MMP9 triggers LYVE1 ectodomain shedding. The shedding was inhibited by MMP9 inhibitors or siRNA knockdown. Notably, MMP9 modulation in hLECs disrupted macrophage adhesion to LECs and impaired transendothelial migration.

Recent studies have highlighted the extracellular matrix glycosaminoglycan HA as a key player in lymphatic attraction, transport, and removal of immunogenic and potentially harmful molecules from tissue interstitium (64, 65). The negatively charged HA polysaccharide facilitates the transmigration and elimination of activated dendritic cells (DCs) and macrophages through interactions with the LV marker LYVE1 (31, 64). To understand how LYVE1 shedding can affect immune cell trafficking, we examined macrophage docking and trafficking across the lymphatic barrier. We found that LYVE1 shedding on LECs disrupted macrophage attachment and hindered their transendothelial migration, an effect that could be reversed with shedding inhibitors. Our results support the idea that LYVE1 shedding and loss of LYVE1-HA binding contribute to impaired immune cell trafficking. These findings in kidney injury build upon and extend the growing literature on the LYVE1/HA axis in other organ systems. In a cutaneous hypersensitivity model, a comprehensive analysis of DC trafficking in wild-type and *Lyve1^–/–^* mice demonstrated that the LYVE1-HA glycocalyx interaction is essential for immune cell docking and entry into primary LVs (64). Disruption of this axis, by either LYVE1-blocking antibodies or genetic deletion of LYVE1, impaired lymphatic entry and lymph node trafficking of CD11c^+^ DCs as well as adoptively transferred bone marrow–derived DCs. Similarly, studies have shown that monocyte-derived macrophages rely on LYVE1 for migration across lymphatic endothelium, a process disrupted by LYVE1-blocking antibodies or hyaluronidase digestion (65–67).

In view of macrophages’ central role (versus DCs) in renal fibrosis (68), we focused on the migration of macrophages. Our study demonstrates that kidney injury leads to LYVE1 shedding, which results in macrophage accumulation around LVs due to impaired macrophage transmigration. These results emphasize the significance of LYVE1 in macrophage-driven tissue remodeling and repair. In particular, LYVE1-mediated macrophage clearance has been crucial in conditions like myocardial infarction, in which LYVE1 deficiency leads to macrophage accumulation and delayed repair (31). Our previous studies have shown that kidney disease impairs lymphatic pumping of renal collecting vessels that predicts reduced tissue clearance (25). The current study expands those observations, showing impairment in the initial steps of macrophage entry into LVs, and the critical role of LYVE1 ectodomain hydrolysis in the LYVE1/HA axis. By diminishing macrophage adhesion and transmigration, LYVE1 hydrolysis may compound lymphatic pumping dysfunction or constitute a regulatory mechanism independent of downstream lymphatic pumping function. Understanding this process is crucial, as it provides insights into how LV dysfunction contributes to macrophage accumulation and persistent inflammation in renal fibrosis; targeting the LYVE1/HA axis could provide a therapeutic approach to mitigate fibrosis and enhance tissue repair in kidney disease and beyond. Macrophage heterogeneity, including resident and infiltrating monocyte-derived populations, is increasingly recognized as a critical factor in fibrosis progression (69). Although our study did not directly distinguish these populations in vivo, loss of HA binding following LYVE1 shedding is expected to impair lymphatic transport of any macrophage subset that depends on HA-LYVE1 interactions for transit. Studies employing lineage tracing or adoptive transfer will be needed to define the relative contributions of distinct macrophage sources to the retention phenotype we observed.

LYVE1 ectodomain shedding is expected to predominantly impact macrophages and DCs, the primary immune cell populations known to utilize an HA glycocalyx for lymphatic entry and transit (30). By contrast, immune cells such as T cells and neutrophil migration through lymphatics use adhesion molecules and receptors that are distinct and independent of HA-LYVE1 interactions. For example, T cells use CD44 binding to macrophage mannose receptor and integrin-mediated mechanisms, while neutrophils employ β_2_ integrin–dependent adhesion and proteolytic remodeling of lymphatic barriers (70, 71). Thus, the functional consequences of LYVE1 shedding for immune cell migration are likely limited to cells dependent on the HA glycocalyx, primarily macrophages, and DCs.

We recognize that our results have some limitations. Although LYVE1 knockout has been linked to impaired immune function and disease progression in mature mouse models, complete removal of the LYVE1 protein may induce complex changes beyond the shedding of the ectodomain. Also, the released ectodomains or intracellular fragments of LYVE1 may retain some functionality, as seen with CD44 (72). In vivo shedding inhibitors and in vitro application of MMP9-targeting siRNAs can produce complex and variable outcomes. Future research should focus on identifying specific MMP9 cleavage sites on LYVE1 and employing point mutations in cell and animal models to better understand the impact of LYVE1 shedding on kidney disease. Additionally, the effects of soluble LYVE1 on both macrophages and renal cells and its precise mechanisms of action need to be further explored. Addressing these questions will be crucial for translating our findings into clinical applications. Our study focused on renal fibrosis, which represents the most consequential sequela of kidney injury. Nevertheless, we recognize that activation and specific pathways leading to LYVE1 ectodomain hydrolysis may differ in various kidney diseases. It is therefore notable that we previously observed similar serum LYVE1 levels in individuals with chronic kidney disease without fibrosis and those with mild fibrosis; only subjects with moderate to severe fibrosis showed a significant increase in LYVE1 (37). This pattern suggests that LYVE1 hydrolysis may be kidney injury stage dependent. In support of this concept, MMP9 increases progressively over the time course in the UUO model (Figure 3K). Future research should explore the regulatory mechanisms and dynamic changes of LYVE1 cleavage in other kidney disease models, including models representing the transition from acute to chronic pathology. Moreover, immunofluorescence localization of MMP9 was not performed because it is a secreted protease with transient extracellular distribution, making cell-associated staining technically challenging and potentially unrepresentative of its functional site of action. In this study, THP-1 monocytes were differentiated into macrophages using PMA, generating cells with an M0-like, non-polarized phenotype. We did not further polarize macrophages into M1 or M2 subtypes, as our objective was to evaluate the general effects of LYVE1 shedding and TGF-β1 on macrophage-LEC interactions. However, macrophage phenotype may influence migratory and adhesion properties, and future studies will be required to determine whether M1- or M2-polarized macrophages exhibit similar patterns in adhesion and transendothelial migration. It is important to note that the direction of macrophage migration in our Transwell assay (apical to basolateral) (73, 74) is opposite to the physiological entry of immune cells into initial LVs, which occurs from the tissue (basolateral side) into the lymphatic lumen (apical side) (75). Our assay was designed to model the egress of macrophages from the interstitial tissue across LECs into LVs, focusing on the molecular mechanisms governing transendothelial migration rather than exact anatomical orientation. While this in vitro system cannot fully recapitulate the complex in vivo environment, it allows dissection of key adhesion and signaling interactions involved in macrophage passage through the lymphatic barrier. We acknowledge this limitation and suggest that future studies using advanced imaging and in vivo models will be necessary to confirm these mechanisms in their physiological context.

In summary, this study demonstrates that elevated MMP9 in the kidney under pathological conditions leads to LYVE1 shedding from LECs, effectively removing macrophage-binding sites. This loss of adhesion prevents macrophages from entering the lymphatic circulation, causing their accumulation in the kidney and contributing to inflammation and fibrosis (Figure 7). Blocking LYVE1 shedding, in turn, reduces macrophage aggregation and disease progression, suggesting an approach for treating kidney fibrosis.

## Methods

### Sex as a biological variable.

Only male mice were used in this study to minimize variability related to hormonal cycles and to maintain consistency across experimental groups. As a result, potential sex-specific differences could not be assessed.

### Animal experiments.

Eight-week-old male C57BL/6 mice weighing 18–20 g (purchased from Shanghai Laboratory Animals Center) were housed with a 1:1 light/dark cycle and acclimated for 7 days before experiments. The well-established models of kidney fibrosis induced by UUO, I/R, and FA were created as previously described (76). Briefly, UUO involved ligation of the left ureter with 5-0 surgical silk followed by sample collection 14 days after surgery. For the FA model, mice were injected i.p. with 250 mg/kg FA dissolved in 300 mM NaHCO_3_, and samples were obtained 28 days later. For the I/R model, ischemia was induced by clamping of the left renal pedicle with non-traumatic microvascular clamps at 37°C for 30 minutes. Seven days later, the right kidney was removed under anesthesia, and 28 days after ischemia, the mice were euthanized and the kidneys were harvested for analysis. Our study exclusively examined male mice. It is unknown whether the findings are relevant to female mice.

In MMP9 supplementation experiments, mice were divided into 4 groups: (a) sham-operated mice (sham group); (b) UUO (UUO group); (c) sham mice treated with shedding inhibitor SB-3CT (10 mg/kg i.p., daily) (MedChemExpress) or GM6001 (10 mg/kg i.p., daily) (MedChemExpress); and (d) UUO mice treated with shedding inhibitor SB-3CT or GM6001 (UUO+SB-3CT or UUO+GM6001).

All mice were sacrificed 14 days after UUO and 28 days after I/R and FA, and the kidneys were harvested for further experiments. All in vivo experiments were conducted in a single cohort of mice. Sham and UUO surgeries, FA and I/R injuries, and inhibitor treatments (SB-3CT, GM6001, UUO+SB-3CT, UUO+GM6001) were performed simultaneously, with *n* = 5 mice per group. As a result, the sham and UUO groups served as shared control groups (Supplemental Figure 1H). This sample size was selected because it provides sufficient statistical power to detect large, biologically relevant effects in this preclinical study while adhering to ethical and practical constraints. For statistical analyses, data from each mouse were treated as individual biological data points. In some instances, the same sham or UUO biological samples were analyzed across multiple Western blots performed in parallel with different experimental comparisons. Because band intensities can vary between blots because of technical factors such as membrane exposure and detection conditions, individual mice occasionally yielded multiple technical measurements for the same protein. In such cases, densitometric values obtained from the same mouse across different blots were averaged to generate a single representative value per animal. These per-animal values were then used for unified statistical analyses and presentation in consolidated bar graphs including all relevant experimental groups (sham, UUO, SB-3CT, GM6001, UUO+SB-3CT, UUO+GM6001) (Supplemental Figure 2, A–J), ensuring accurate representation of the shared-control design without duplication of biological samples.

Mice were to be excluded only if the fibrotic model failed or if they died before the endpoint. No mice were excluded in the experiments. A cage of mice was randomly selected to be assigned to the normal or disease or treatment group, and the dosing order was changed every day during the treatment to minimize the effect of bias. Analgesics were administered every 12 hours within the first 48 hours after surgery, and the mice’s activity levels and overall condition were monitored daily.

### Cell culture.

The human lymphatic endothelial cell (hLEC) line (National Collection of Authenticated Cell Cultures, Shankghai, China) was cultured with an endothelial cell medium kit (ScienCell). hLECs were treated with 10 ng/mL transforming growth factor-β1 (TGF-β1) (R&D Systems) with or without shedding inhibitors (GM6001 and SB-3CT: 10 mM; MedChemExpress) for 48 hours. Small interfering RNA (siRNA) was used in hLECs to induce MMP9 silencing, and protein was collected after 48 hours to measure the knockdown effect.

Human myeloid leukemia mononuclear (THP-1) cell line (National Collection of Authenticated Cell Cultures) was cultured in 1640 medium supplemented with 10% fetal bovine serum, 10% penicillin/streptomycin (New Cell and Molecular Biotech), and 1% β-mercaptoethanol (MilliporeSigma). All cells were cultured at 37°C, 5% CO_2_. THP-1 cells were differentiated with 100 nM phorbol 12-myristate 13-acetate (PMA; MedChemExpress) for 24 hours.

### Immunohistochemistry and immunocytochemistry.

Kidneys were fixed overnight in 4% paraformaldehyde, embedded, and then cut into 3-μm sections. After deparaffinization, sections were subjected to antigen retrieval with pH 8.0 solution at 95°C for 20 minutes and blocked with 10% goat serum for 60 minutes at room temperature. Tissue was incubated with primary antibodies against podoplanin (PDPN; Thermo Fisher Scientific, 14-5381-82), LYVE1 (Thermo Fisher Scientific, PA116635, MA5-35489), F4/80 (Abcam, ab300421), and MMP9 (Abcam, ab228402) overnight at 4°C. Goat anti-rabbit secondary antibody (Abcam) in blocking serum was added for an hour of incubation at room temperature. Negative controls were performed with rabbit IgG (Merck Millipore) instead of primary antibody to verify the antibody specification. F4/80-stained kidney sections were imaged under identical exposure and magnification settings. Images were coded by an independent investigator so that the observer performing the quantification was blinded to group allocation. The percentage of F4/80^+^ area within the cortical and medullary regions was quantified using ImageJ software (NIH) with a fixed threshold applied to all images.

For cultured cells, 14 mm tissue culture–treated round cell dishes (WHB Scientific) were used for seeding. Cells were fixed with 4% paraformaldehyde, membrane-permeabilized with 0.1% Triton X-100, blocked with 10% goat serum, and then incubated sequentially with primary antibodies (Cyto-LYVE1, Abcam; full-length LYVE1, Novus) and immunofluorescence-labeled secondary antibodies as described above.

### Western blot.

Cells and tissues were lysed with RIPA buffer containing 1% protease inhibitor (Beyotime). Proteins were separated in SDS-polyacrylamide gels and moved onto PVDF membranes with wet blotting (Bio-Rad) for 40 minutes at 400 mA. Blots were subsequently blocked in 5% skimmed milk and incubated with primary antibodies against PDPN (Thermo Fisher Scientific, 14-5381-82), fibronectin (FN; Abcam, ab2413), collagen I (Col I; Abcam, ab138492), smooth muscle actin (Abcam, ab124964), LYVE1 (Abcam, ab218535; Bioss, bs-1311R), F4/80 (Abcam, ab300421), and MMP9 (Abcam, ab228402) overnight at 4°C. HRP-labeled goat anti-rabbit (or anti-mouse) IgG (H+L) (Beyotime) was used as the secondary antibody. For signal detection, high-performance chemiluminescent developer (EpiZyme) was used according to the manufacturer’s protocol. The relative density of bands was calculated using ImageJ (version 1.8.0, NIH).

### Enzyme-linked immunosorbent assay.

After 48 hours of treatment, the medium was harvested and centrifuged, and the supernatant was collected for further determination. The protein level of soluble LYVE1 in a conditioned medium was detected with a commercial enzyme-linked immunosorbent assay (ELISA) kit (Cusabio) according to the instructions.

### Gelatin zymography.

The gelatin zymography was used to determine the presence of MMP9 as previously described (77). Cells were cultured in serum-free conditioned medium, and the retained supernatant was dissolved in native gel sample loading buffer (New Cell and Molecular Biotech, WB3002), loaded on 10% polyacrylamide gel (with 1% SDS), and separated at a constant 150 V voltage. The gels were transferred to 2% Triton X-100 at 37°C for 30 minutes to remove SDS, and submitted to an incubation solution containing 0.05 M Tris/HCl and 5 mM CaCl_2_ at 37°C for 24 hours. Relative expression of MMP9 was visualized by staining with 1% Coomassie brilliant blue R-250 (EpiZyme). Total protein in the supernatant was shown by Coomassie brilliant blue staining.

### Macrophage adhesion assay.

After THP-1 cells were differentiated into macrophages, cells were harvested and labeled with 5 μM CFDA-SE at 37°C for 1 hour in the dark. After stopping of the labeling program with pre-cold PBS and washing with serum-free medium 2 times, macrophages were plated in wells with hLECs (incubation with or without TGF-β1 or MMP9 inhibitors or siRNA for 48 hours). After 30 minutes of coculture, plates were rinsed twice with PBS to remove unadhered macrophages, and the number of fluorescent cells was counted under a microscope in 5 randomly selected fields of view for each well. The sum was calculated as the number of adherent cells per well, with 3 replicate wells per group.

### Macrophage transendothelial assay.

hLECs were cultured with or without TGF-β1 or MMP9 inhibitors for 48 hours and then seeded into Transwell inner inserts (Corning, 3422). After the cells formed a confluent monolayer, CFDA-SE–labeled macrophages were added to the inner insert. The upper chamber was loaded with serum-free medium, and the lower chamber loaded with 20% serum medium to promote macrophage migration. After 6 hours, the lower membrane was removed and immobilized, and the cells on the upper membrane were wiped with a cotton swab. The number of fluorescent cells was then counted under a microscope as described above. The culture medium of the lower chamber was collected and analyzed for cell shedding using a fluorescent enzyme marker visualized under a microscope.

### Macrophage chemotaxis assay.

Macrophage migration was evaluated using Transwell inserts (Corning, 3422). Cells were seeded in the upper chamber, and medium with or without TGF-β1 was placed in the lower chamber. After 12 hours, non-migrated cells were removed, and migrated macrophages on the lower membrane surface were fixed, stained with 0.2% (wt/vol) crystal violet (Thermo Fisher Scientific, B21932.36), and quantified microscopically.

### Statistics.

Data analysis was conducted using a double-blind approach by a separate person who was unaware of the group assignments. Statistical analysis was performed using GraphPad Prism 7.0 (GraphPad Software). Data passed normality and equal variance tests and were compared using 2-tailed *t* tests (2 groups) or 1-way ANOVA followed by Dunnett’s or Bonferroni’s multiple-comparison test (multiple groups). Data were expressed as mean ± SD, and *P* values less than 0.05 were considered statistically significant.

### Study approval.

All experiments involving animals were approved by the Institutional Animal Care and Use Committee of Shanghai Tongji University Affiliated Tongji Hospital (approval TJ-HB-LAC-2023-44).

### Data availability.

All data are presented in the article. There are no genetic, proteomic, or transcriptomic data, and no specific codes or algorithms are used in this article. All data and other materials are available on reasonable request. Values for all data points in graphs are reported in the Supporting Data Values file.

## Author contributions

JL, CY, and VK designed the study. JL and YL worked on animal experiments. JL and SZ worked on in vitro experiments. WZ worked on data analysis. VK, JZ, and HY provided technical support. AK, VK, and CY did critical editing.

## Funding support

This work is the result of NIH funding, in whole or in part, and is subject to the NIH Public Access Policy. Through acceptance of this federal funding, the NIH has been given a right to make the work publicly available in PubMed Central.

- NIH grants (RO1DK135764J, PO1HL116263, AHA 25POST1373711, and AHA 25CDA1450556).
- National Natural Science Foundation of China (82470764 and 82170696).
- “Science and Technology Innovation Action Plan” of Shanghai Science and Technology Commission (23Y11908900).
- Shanghai Shenkang Hospital Development Center Project (SHDC12022104).
- Shanghai Pujiang Project (25PJD115).
- Clinical Research Project of Tongji Hospital of Tongji University (grant ITJ(ZD)2201).

## Supplementary Material

Supplemental data

Unedited blot and gel images

Supporting data values

## Version 1 01/26/2026

In-Press Preview

## Version 2 03/09/2026

Electronic publication
