# Supplementary material for: LYVE1 ectodomain shedding blunts lymphatic transmigration and clearance of macrophages during kidney injury
Source: JCI Insight. 2026 Jan 22;11(5):e195176. doi: 10.1172/jci.insight.195176 (PMC13041691; doi:10.1172/jci.insight.195176)
Supplement: Supplemental data [file jciinsight-11-195176-s277.pdf]

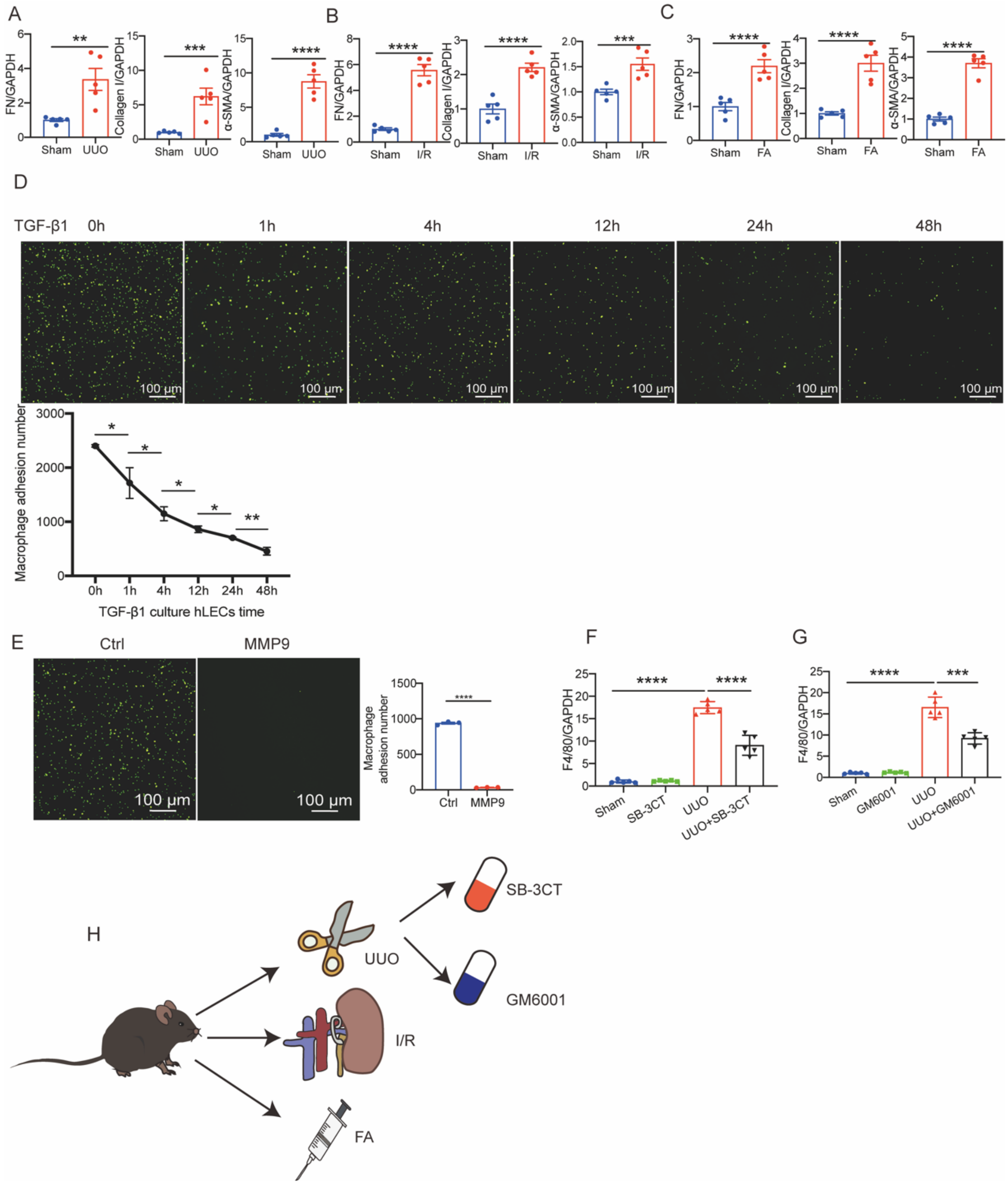

Supplementary Figure 1. (A-C) Bar graph of western blot of FN, Collagen I and α-SMA in UUO(A), I/R(B), FA(C). **(D)** Macrophage adhesion to hLECs progressively decreased with increasing duration of TGF-β1 stimulation. **(E)** Direct MMP9 intervention in hLECs markedly reduced macrophage adhesion. **(F, G)** Bar graph of western blot of F4/80 in UUO with SB-3CT**(F)** or GM6001**(G)**. n=5 per group for animal experiments and n=3 for cell experiments, statistics used included ANOVA analysis. **(H)** Schematic overview of the experimental design and shared-control structure.All in vivo experiments were conducted using a single cohort of mice, with n=5 animals per group. Sham and UUO surgeries, folic acid (FA) injection, ischemia-reperfusion (I/R) injury, and inhibitor treatments (SB-3CT, GM6001, UUO+SB-3CT, UUO+GM6001) were performed simultaneously. Because these interventions were carried out in parallel, the sham and UUO groups functioned as shared controls across multiple analyses and figures in the study.

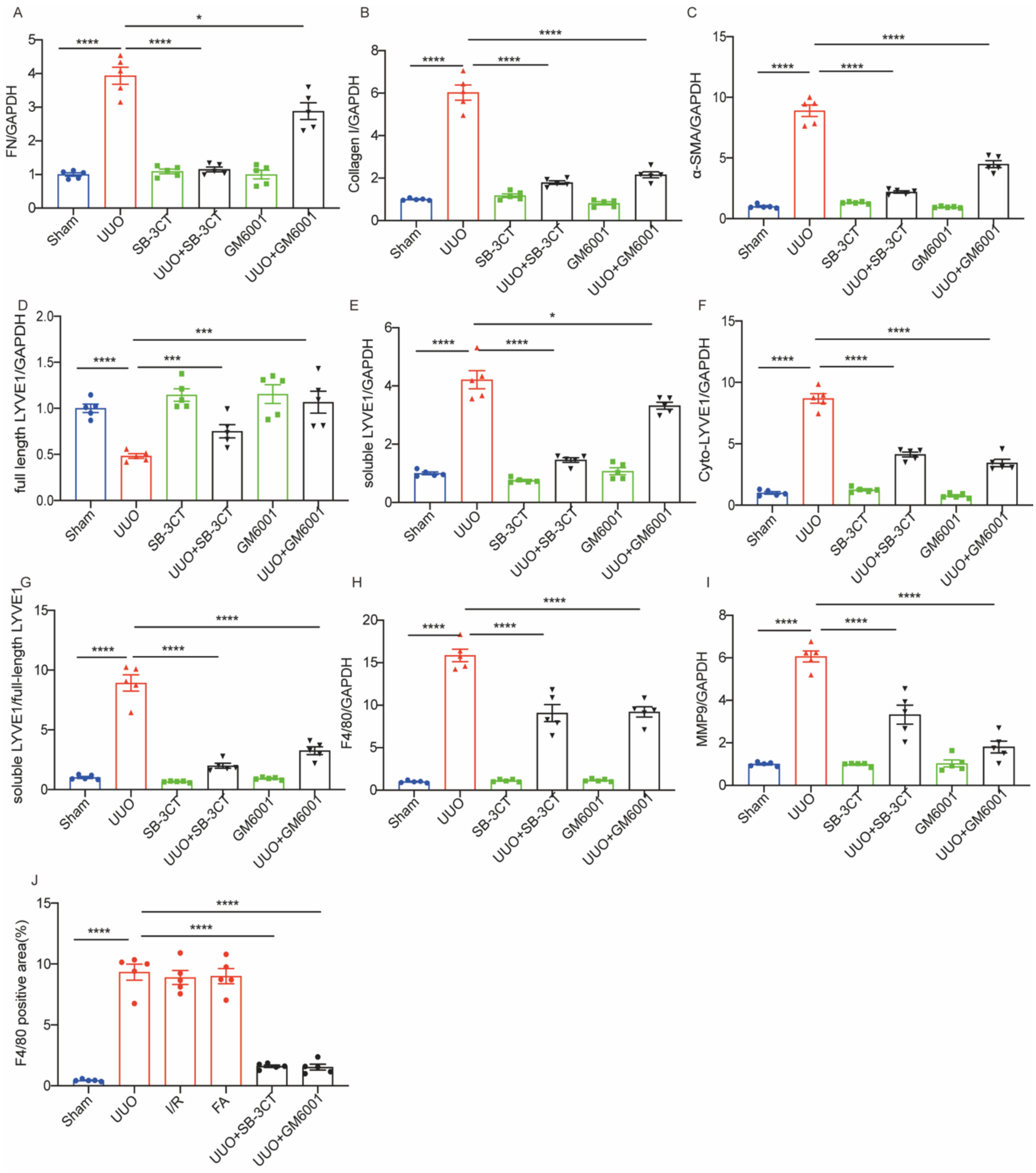

Supplementary Figure 2. (A-I) Consolidated statistical analysis of Western blot quantification using shared sham and UUO control groups. To ensure transparency regarding the shared-control design, Western blot densitometry values from each mouse (n=5 per group) were reanalyzed together across all relevant groups. The unified bar graphs include sham, UUO, SB-3CT, GM6001, UUO+SB-3CT, and UUO+GM6001, based on the same set of sham and UUO biological samples used throughout the study. Each point represents an individual mouse. Statistical analysis was performed using one-way ANOVA.(J) Consolidated statistical analysis of F4/80 positive area using shared sham and UUO control groups. Each point represents an individual mouse. Statistical analysis was performed using one-way ANOVA.
