# Supplementary material for: LYVE1 ectodomain shedding blunts lymphatic transmigration and clearance of macrophages during kidney injury
Source: JCI Insight. 2026 Jan 22;11(5):e195176. doi: 10.1172/jci.insight.195176 (PMC13041691; doi:10.1172/jci.insight.195176)
Supplement: Unedited blot and gel images [file jciinsight-11-195176-s278.pdf]

Full unedited gel for Figure 1A

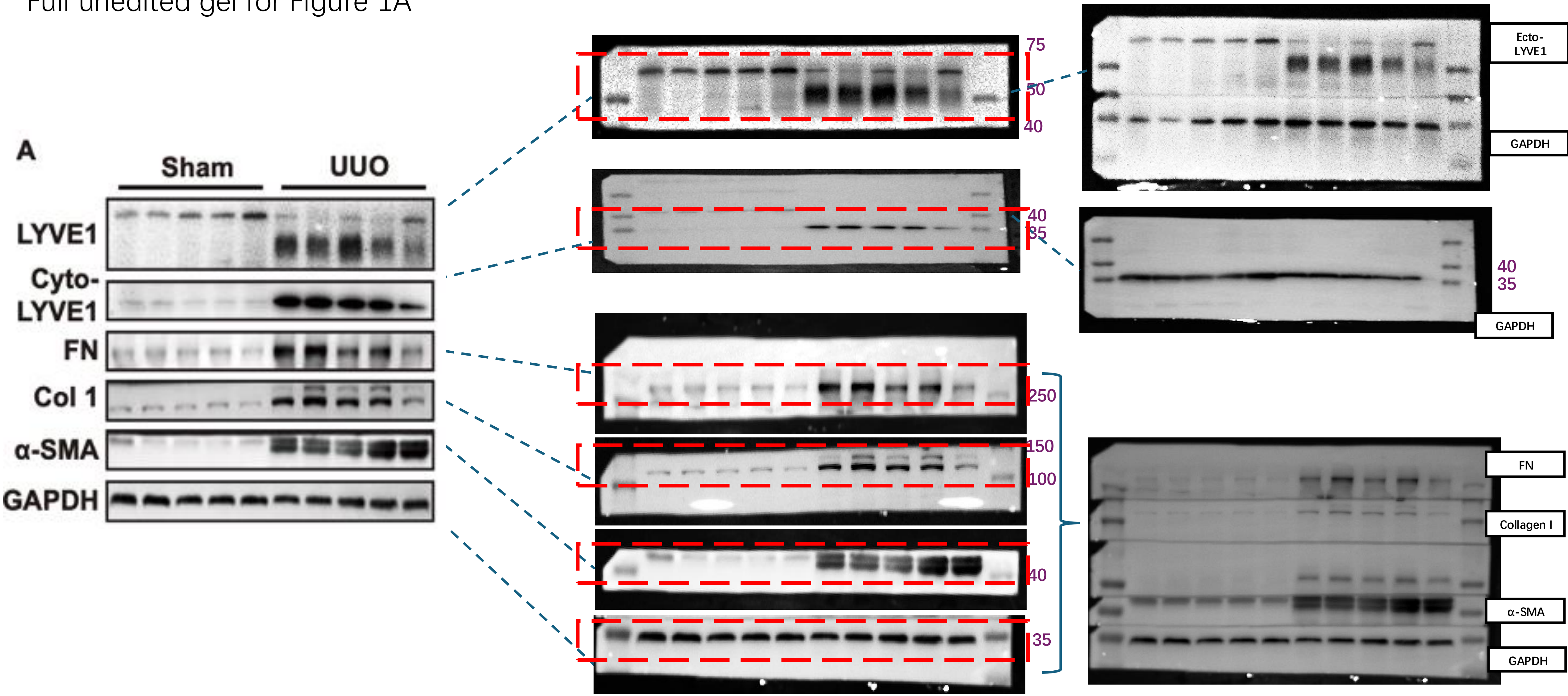

Full unedited gel for Figure 1B

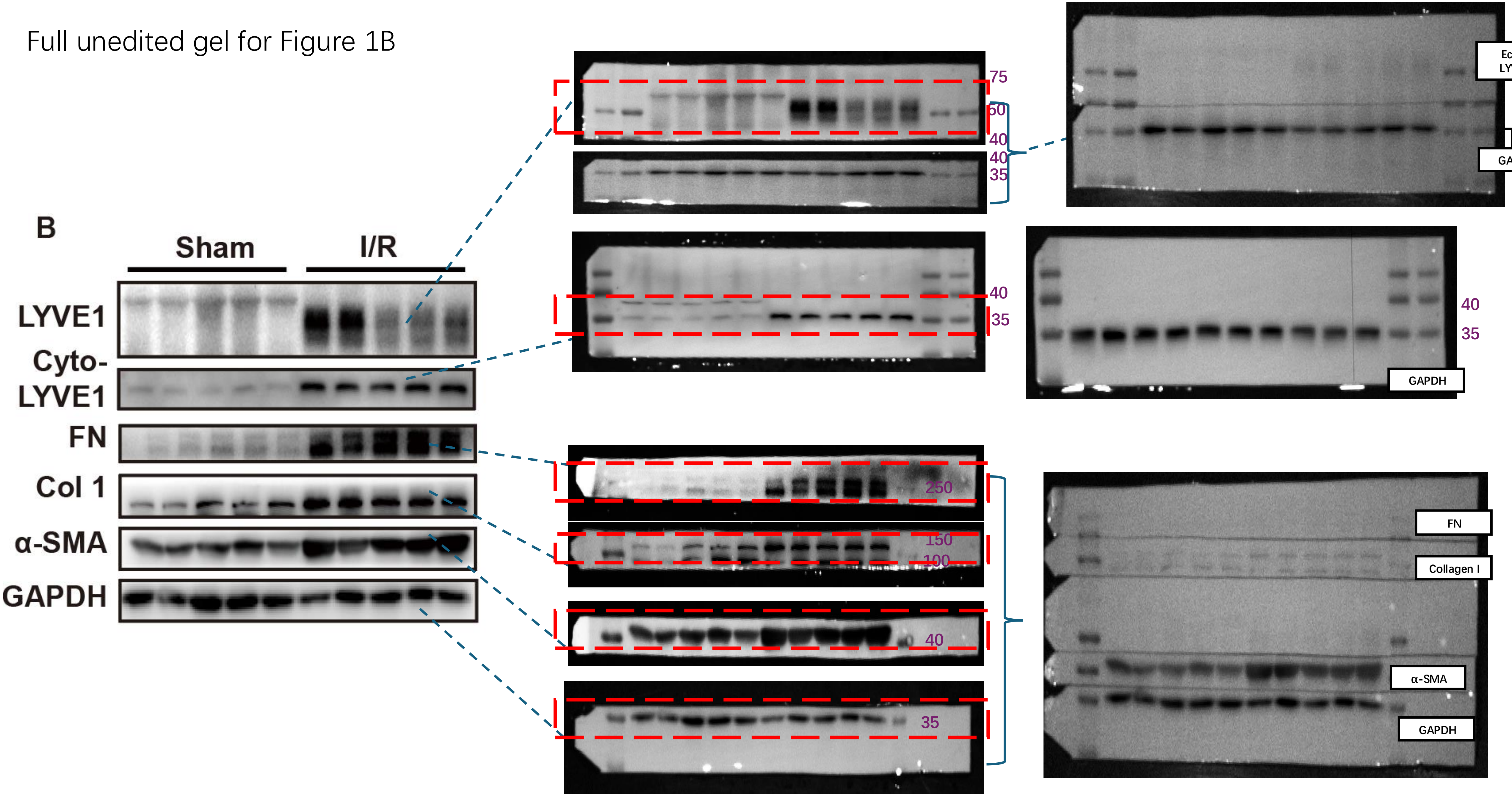

Full unedited gel for Figure 1C

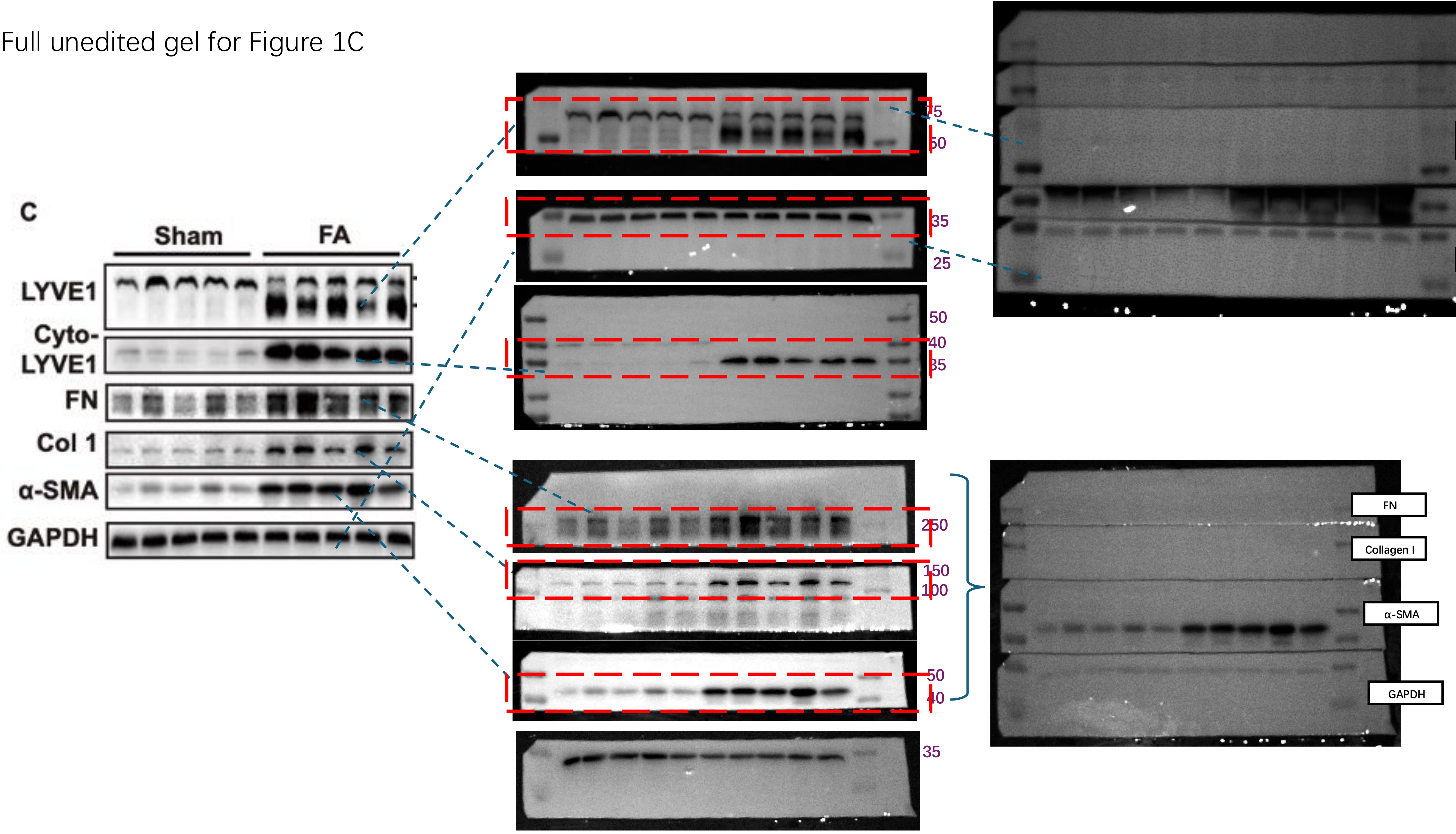

Full unedited gel for Figure 1G

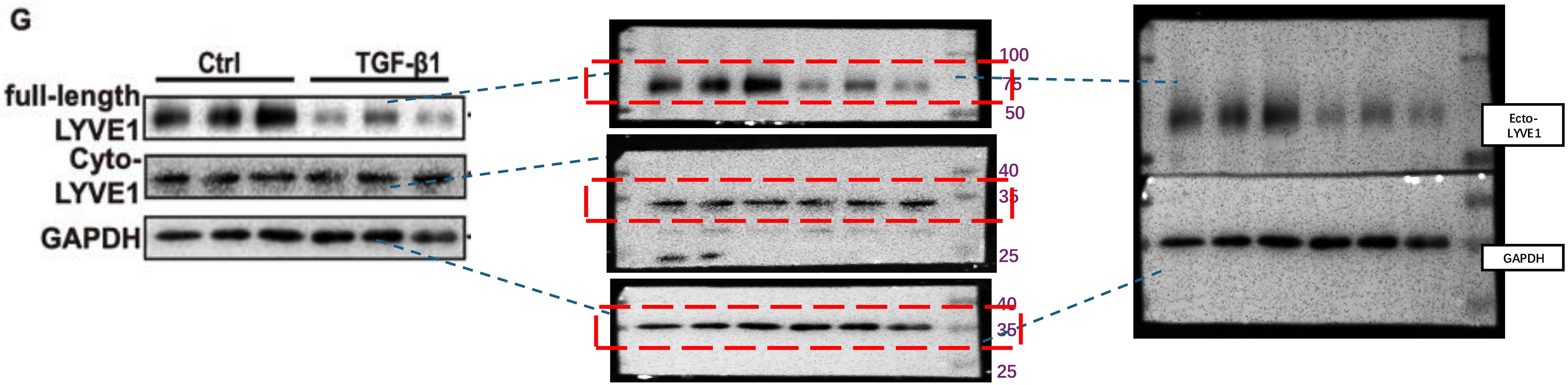

Full unedited gel for Figure 2B

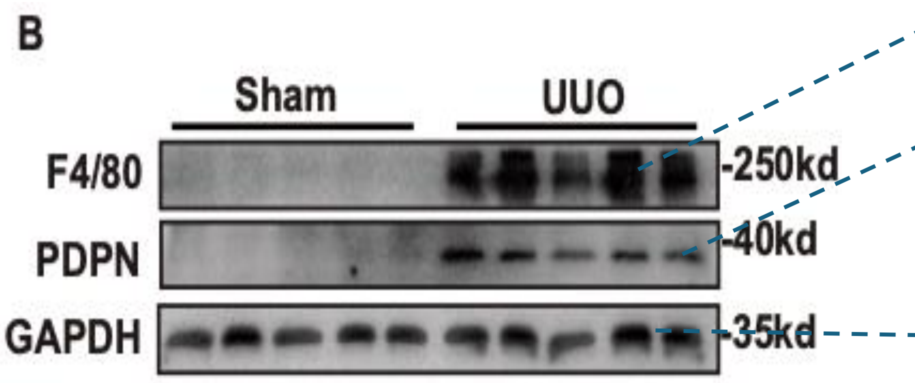

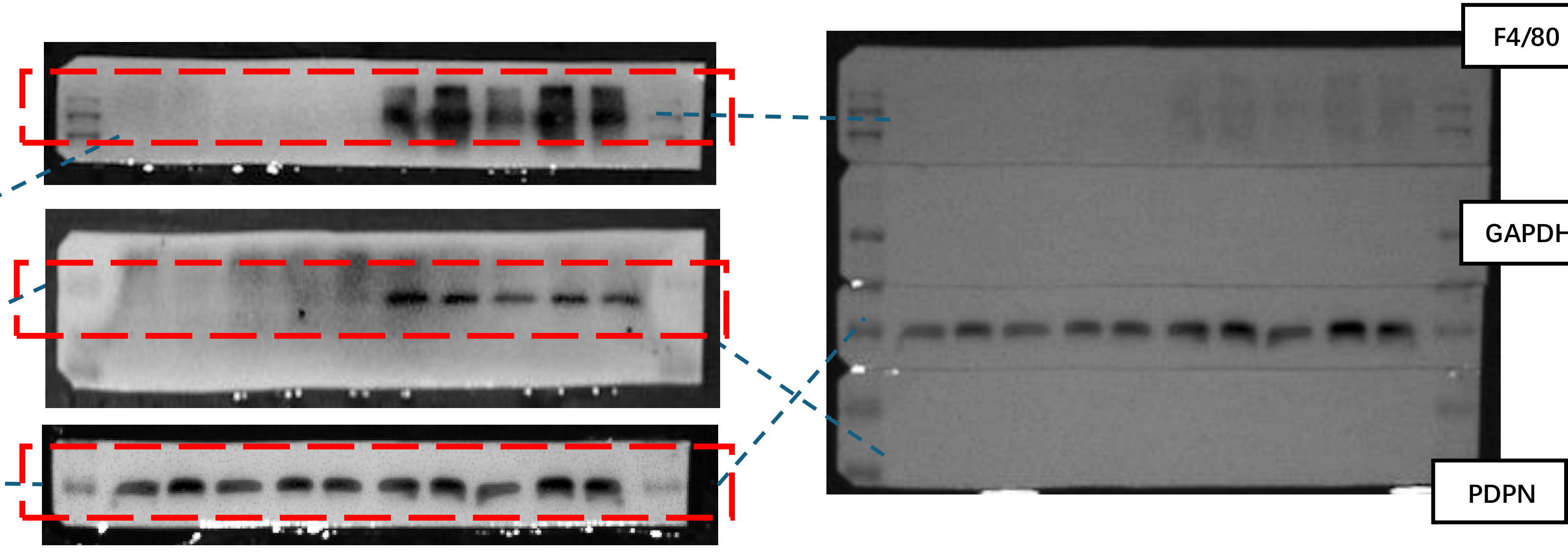

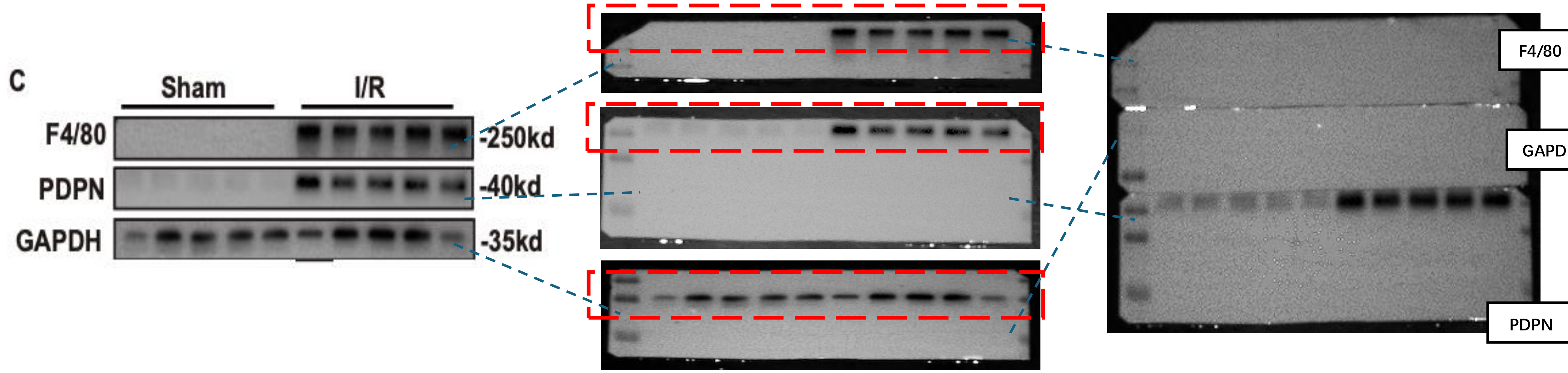

Full unedited gel for Figure 2D

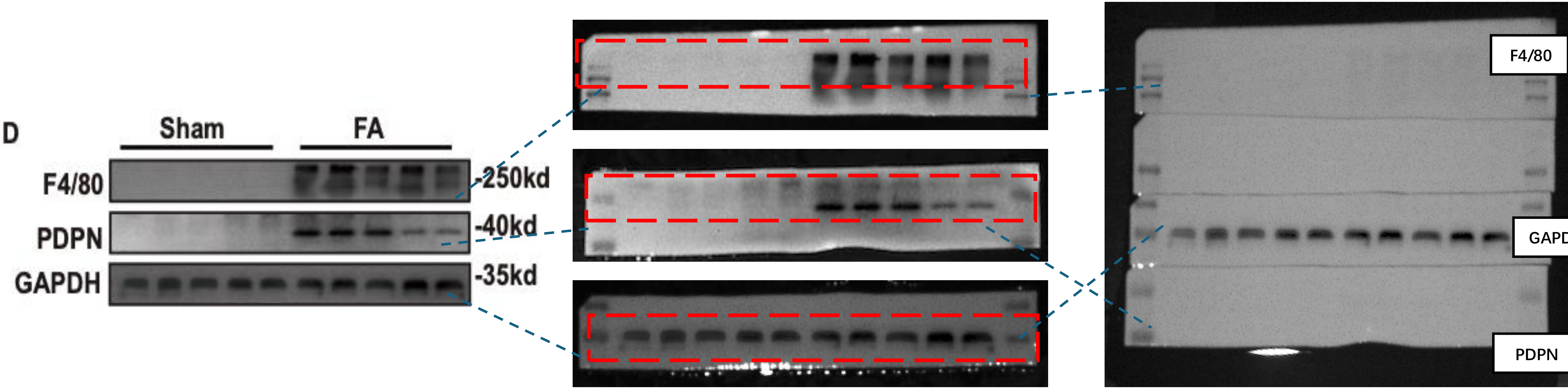

Full unedited gel for Figure 3B

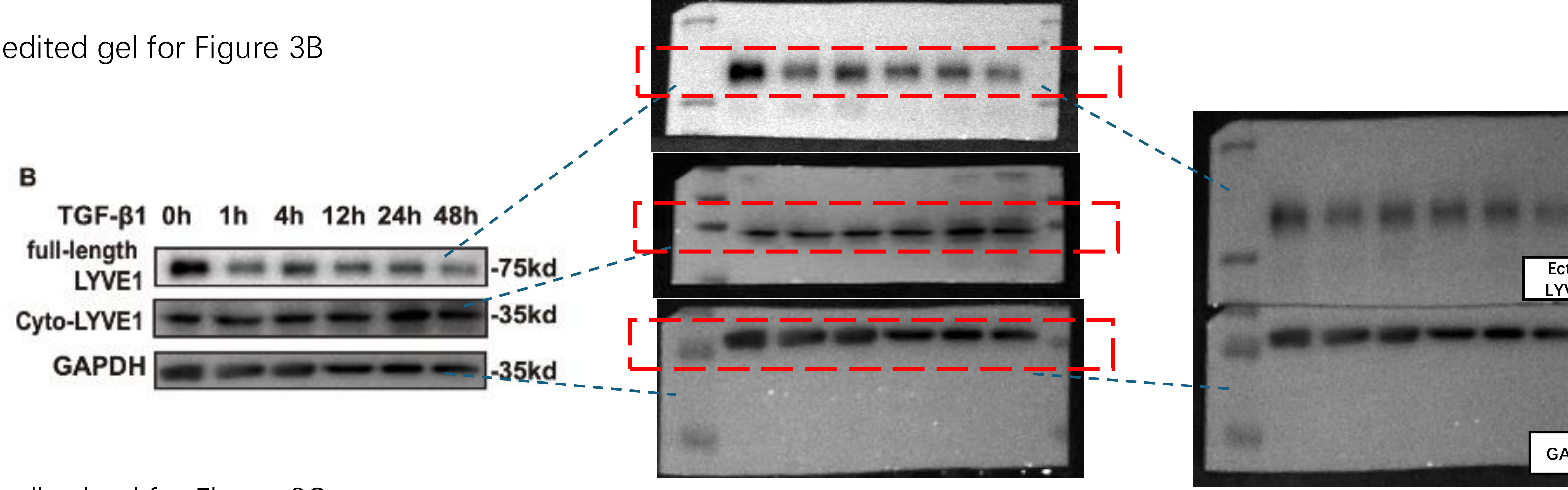

Full unedited gel for Figure 3C

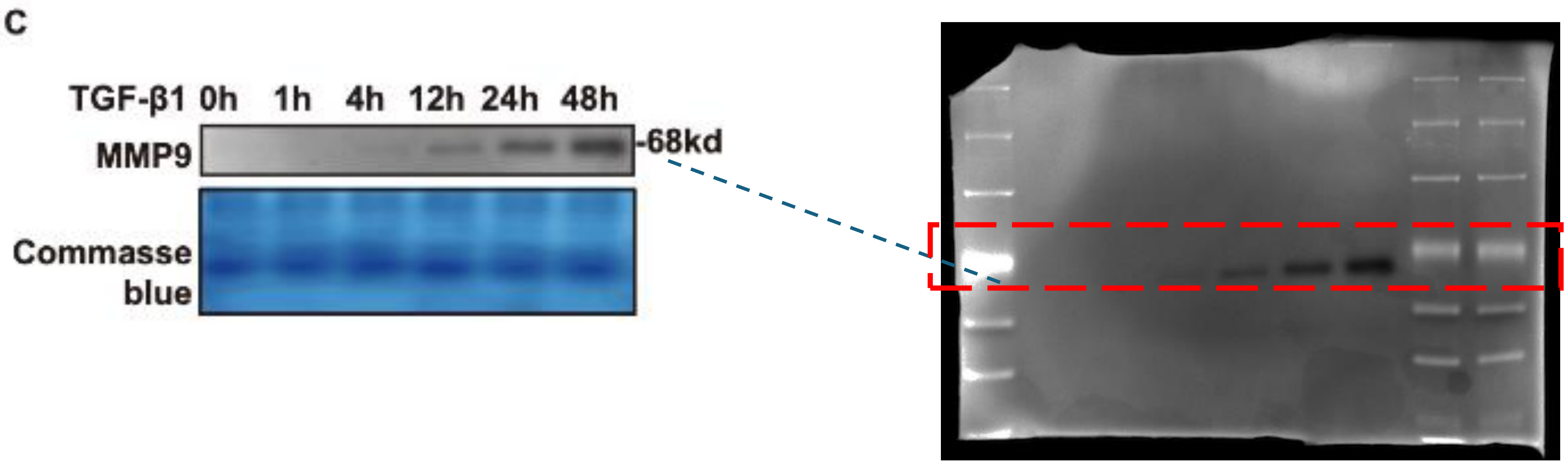

Full unedited gel for Figure 3D

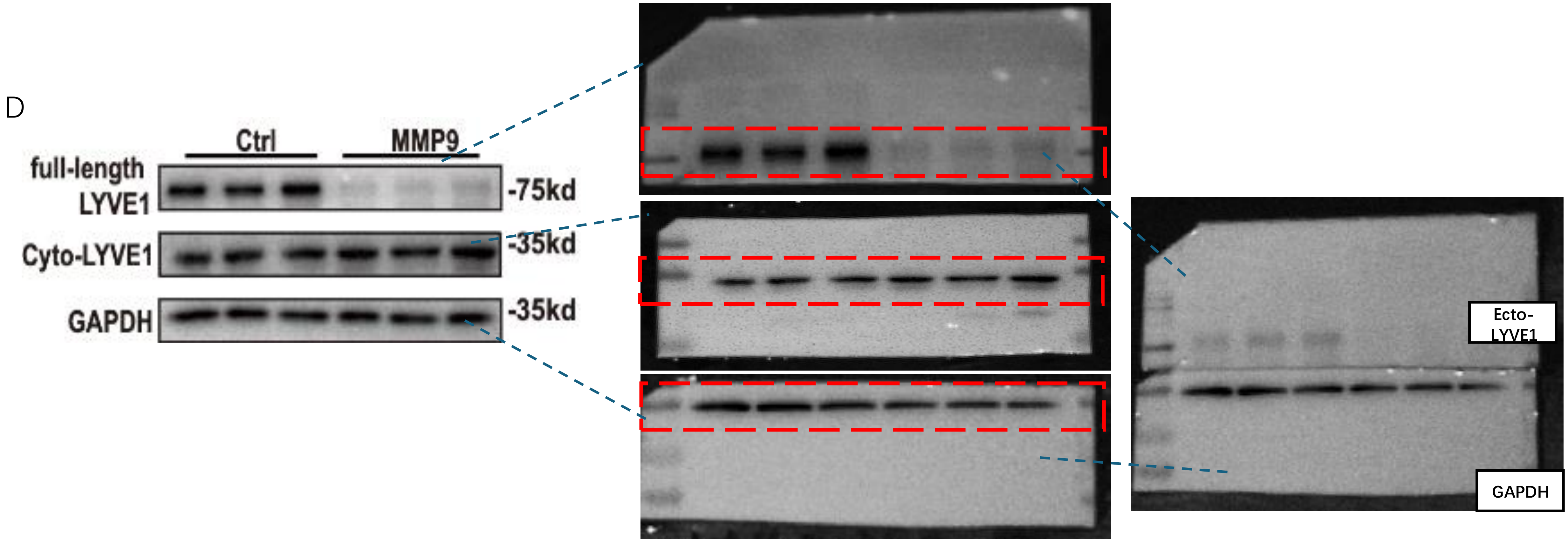

Full unedited gel for Figure 3H

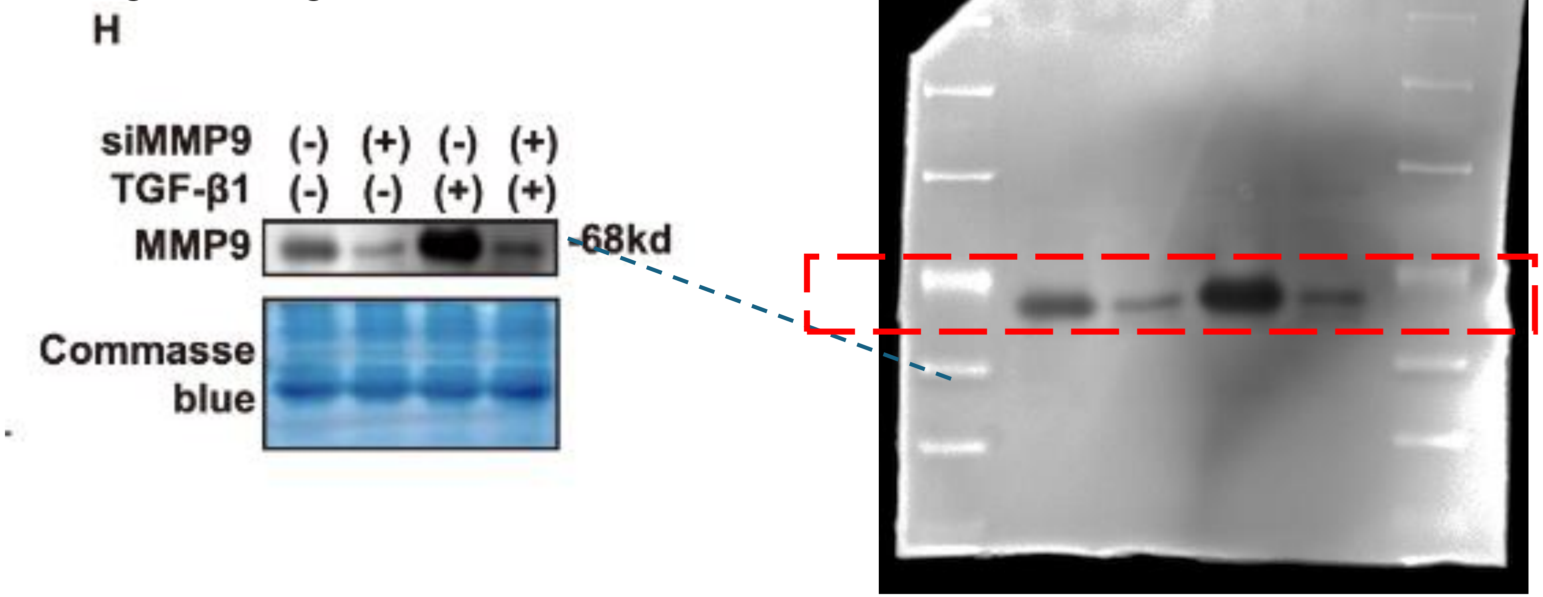

Full unedited gel for Figure 3I

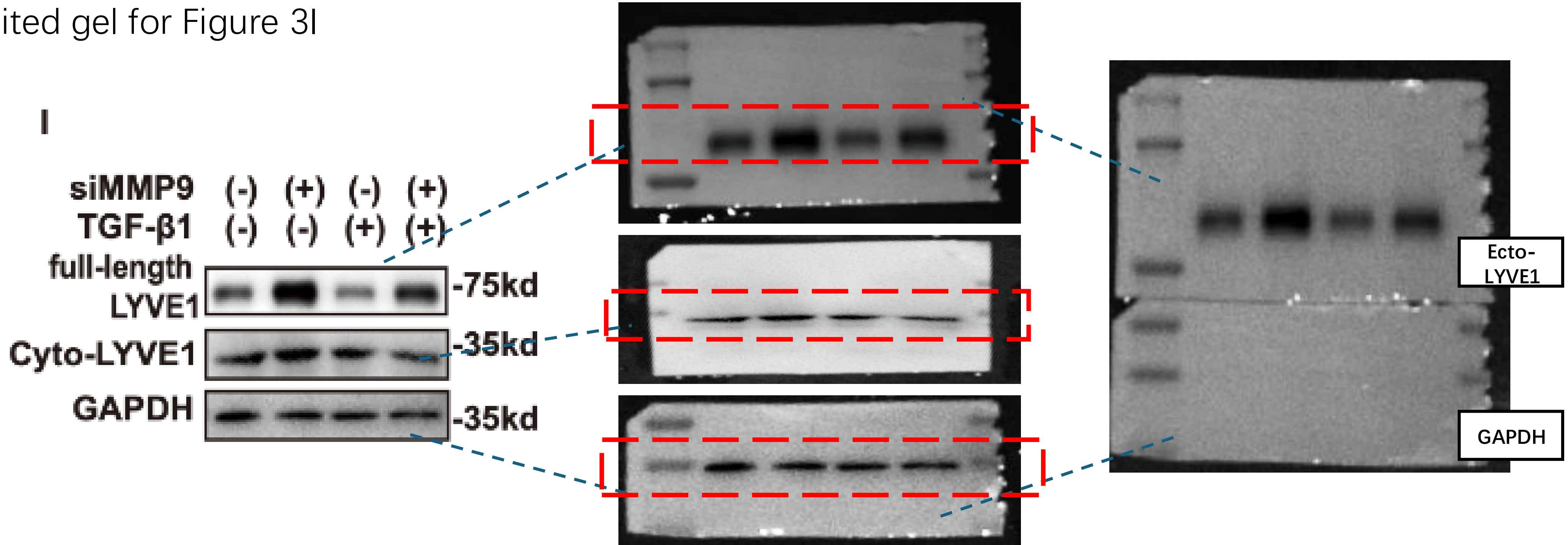

Full unedited gel for Figure 3L

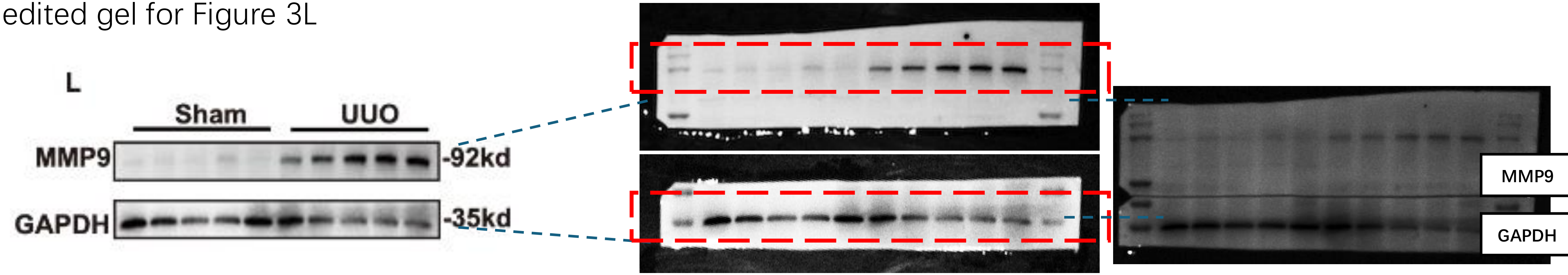

Full unedited gel for Figure 3M

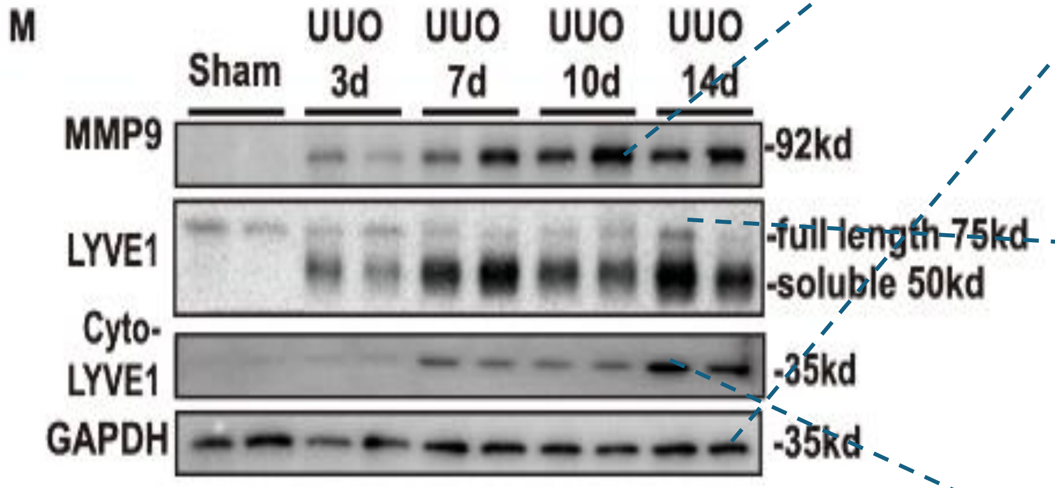

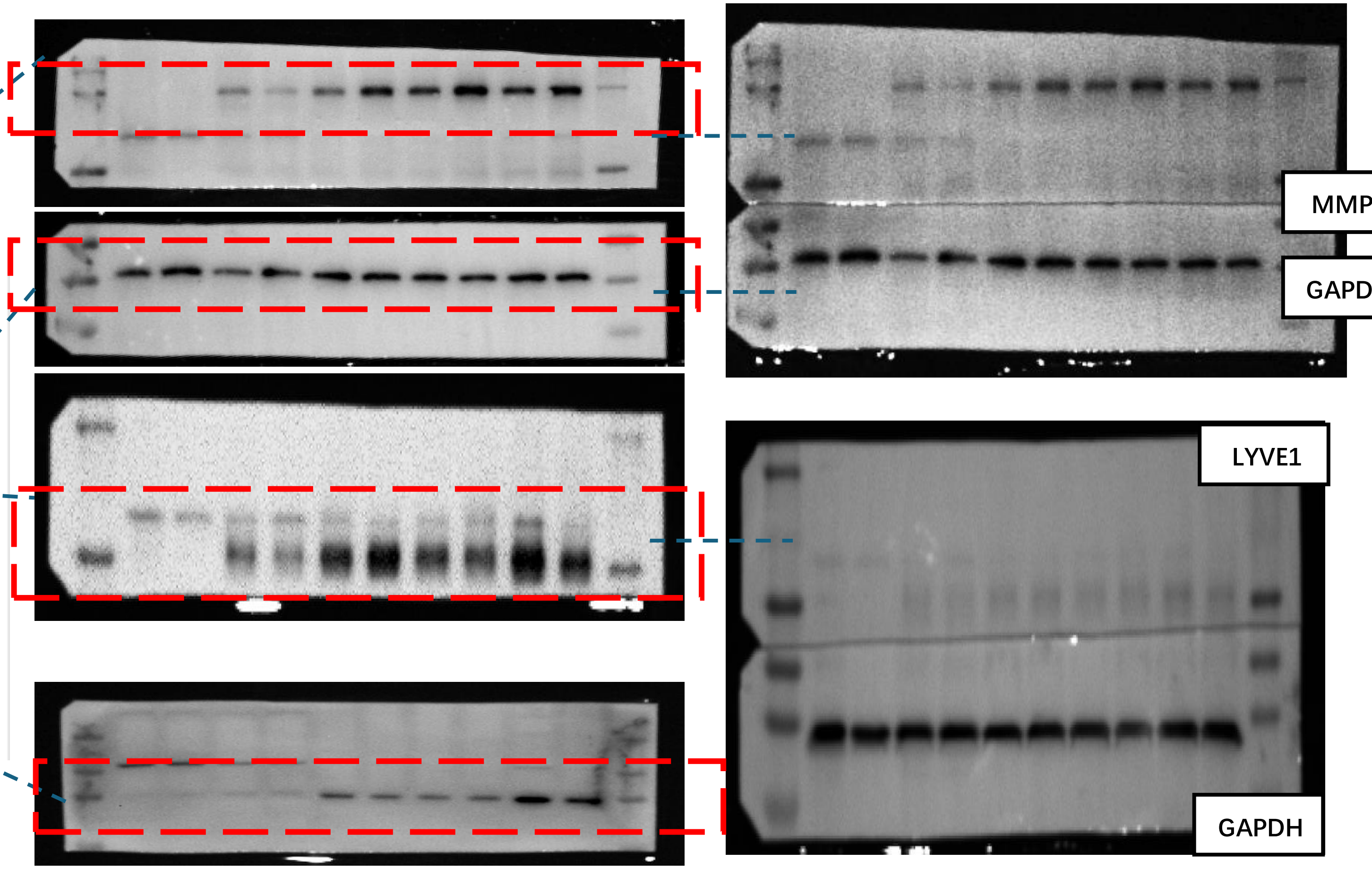

Full unedited gel for Figure 4A

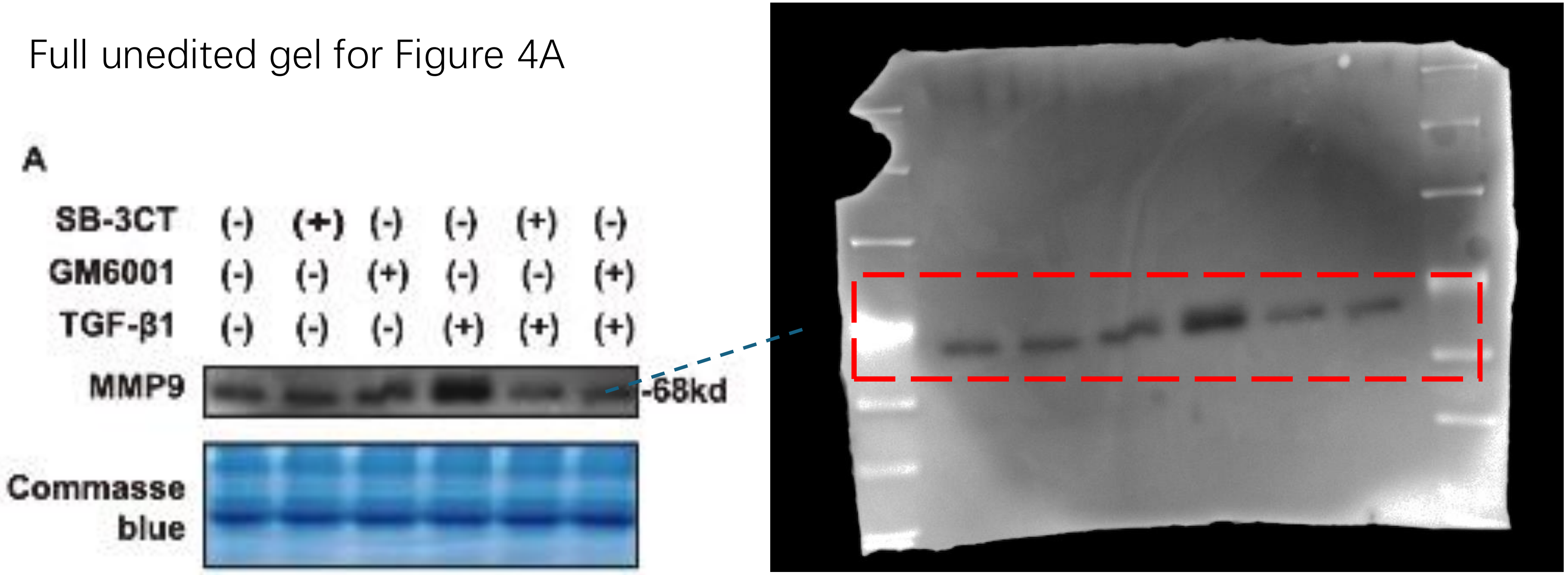

Full unedited gel for Figure 4B

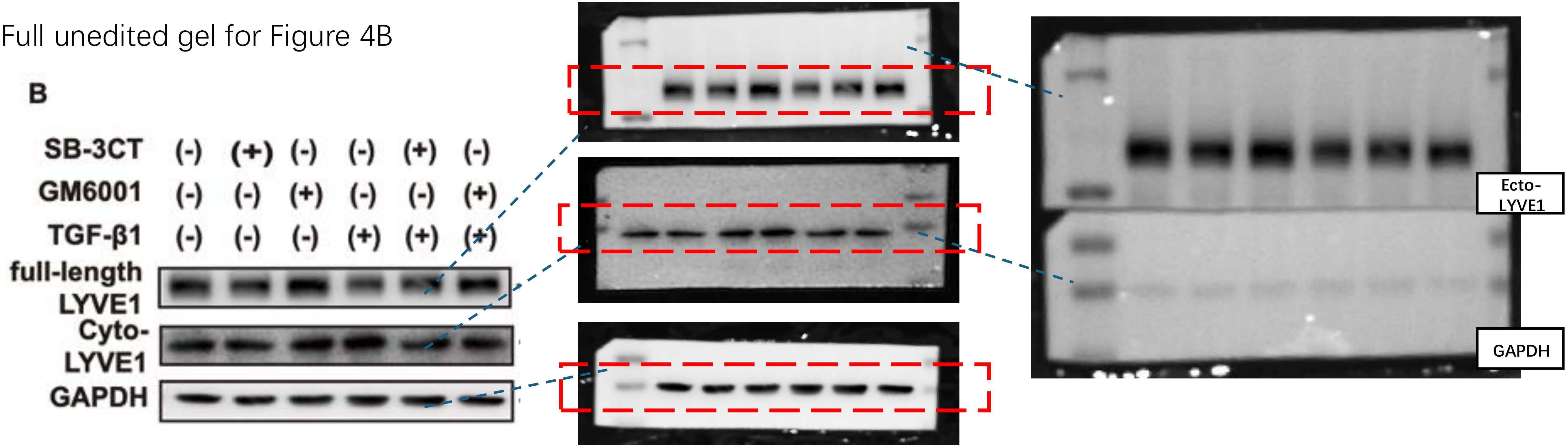

Full unedited gel for Figure 4E

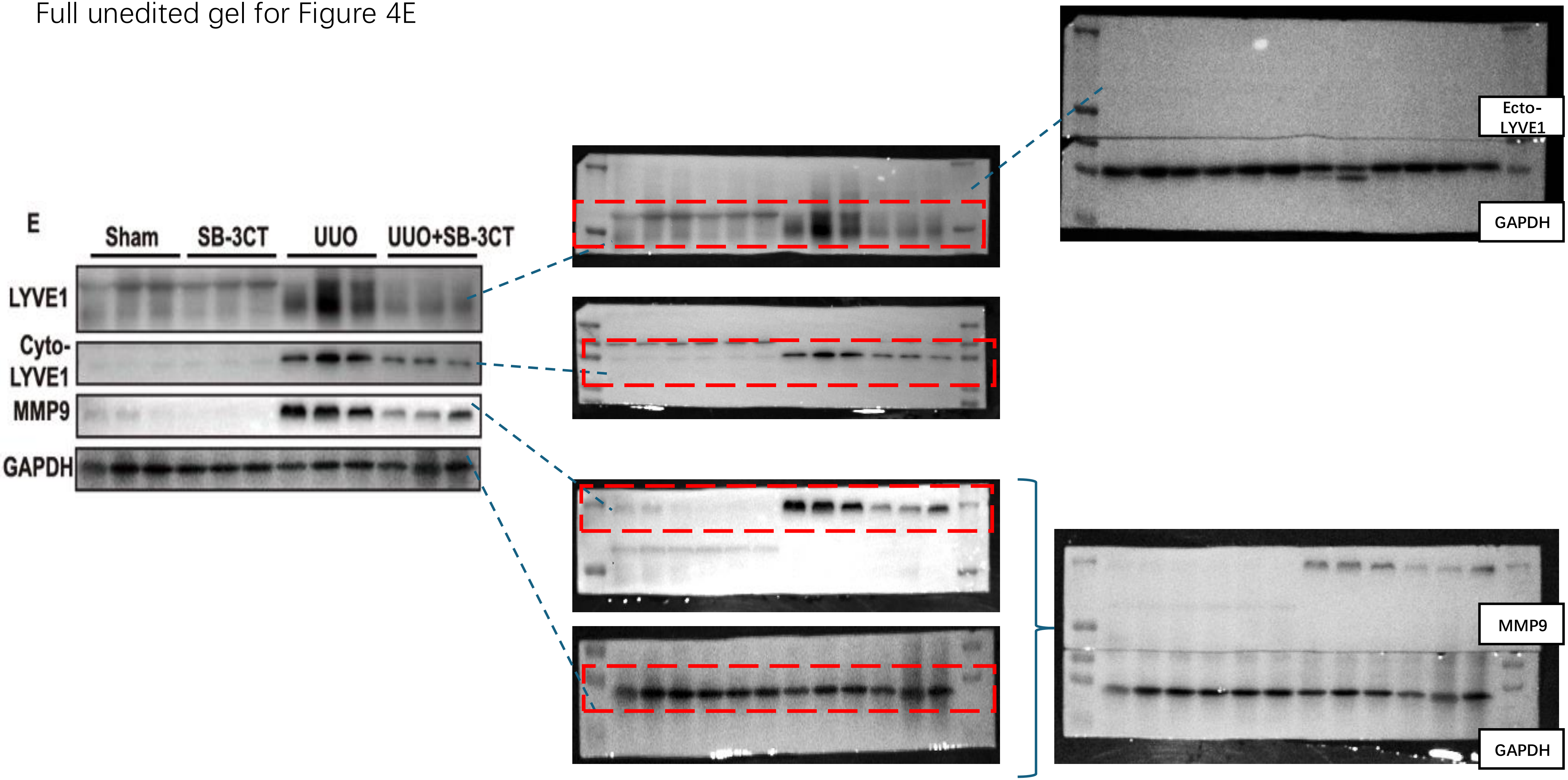

Full unedited gel for Figure 4F

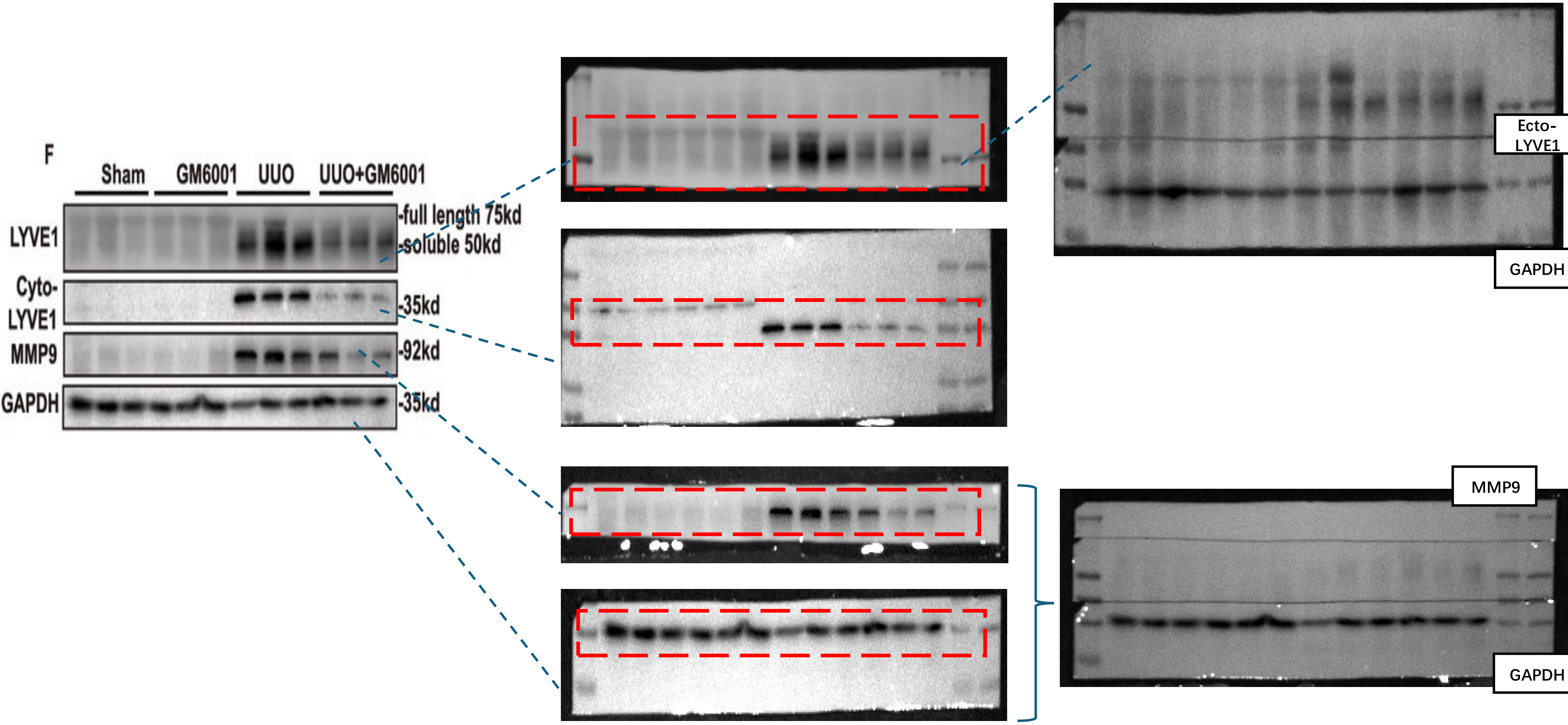

Full unedited gel for Figure 5G

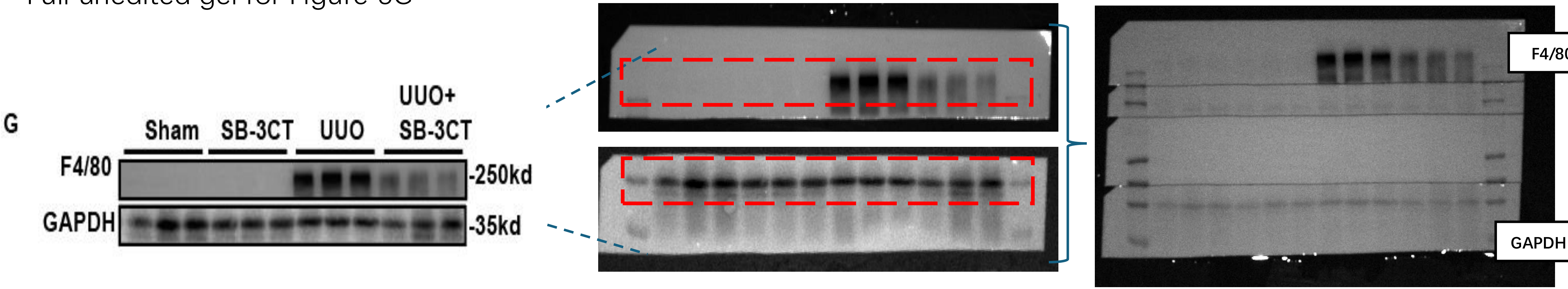

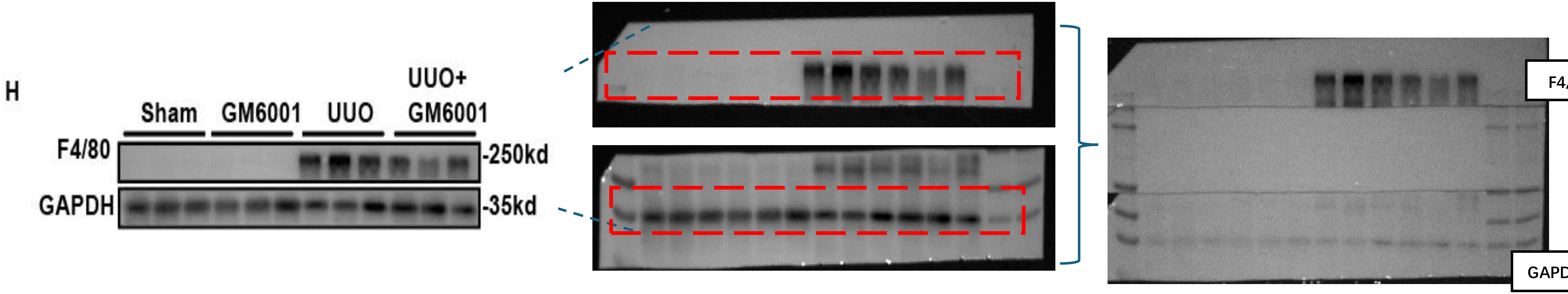

Full unedited gel for Figure 6A

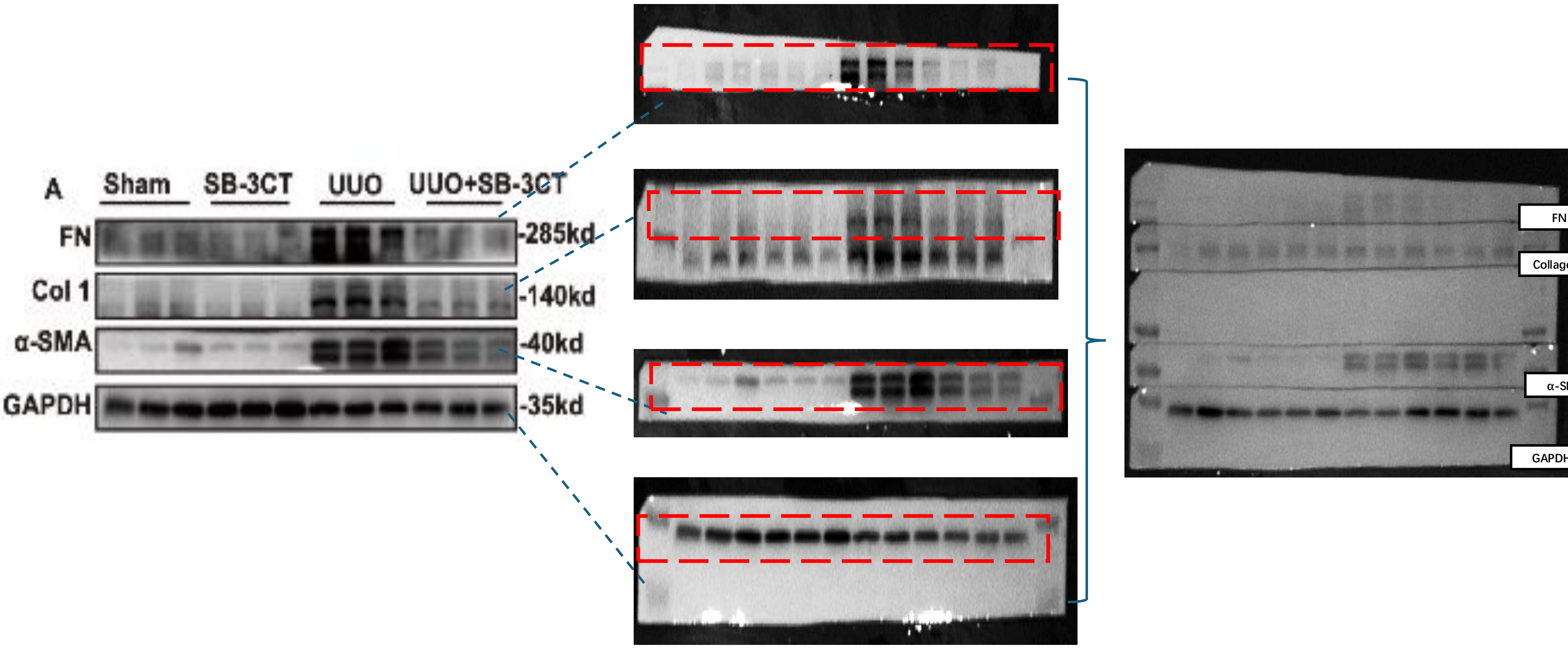

Full unedited gel for Figure 6B

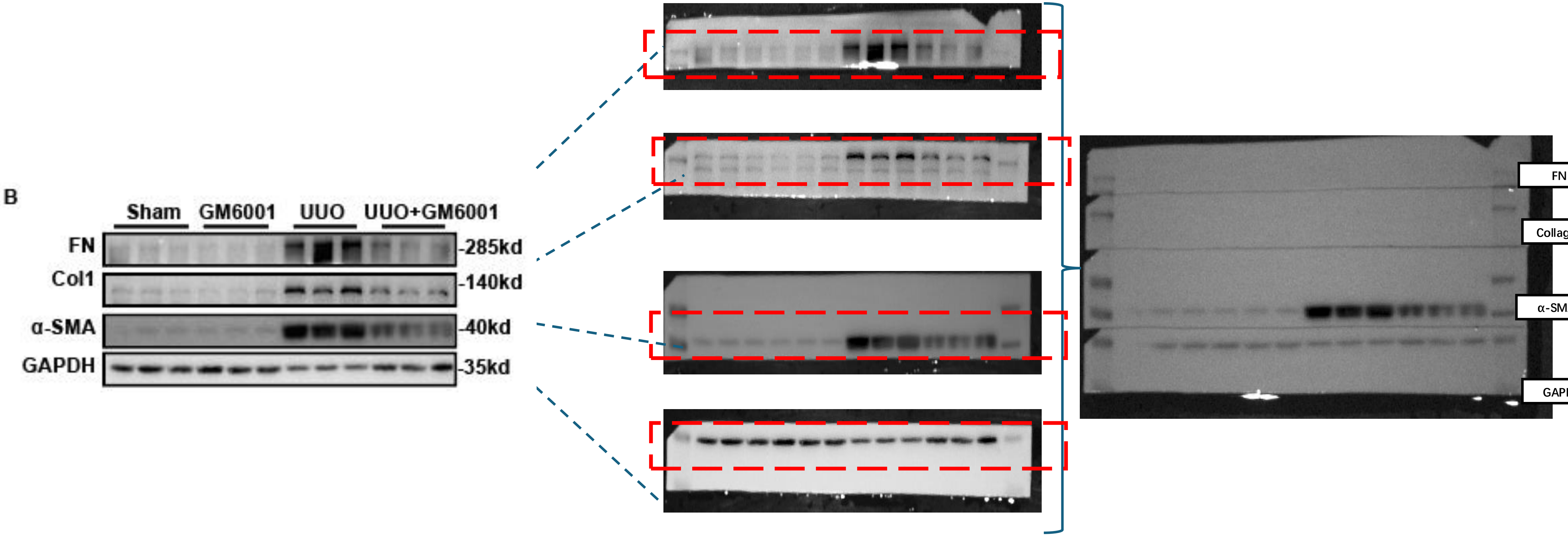

Additional blot for UUO+SB-3CT/UUO+GM6001

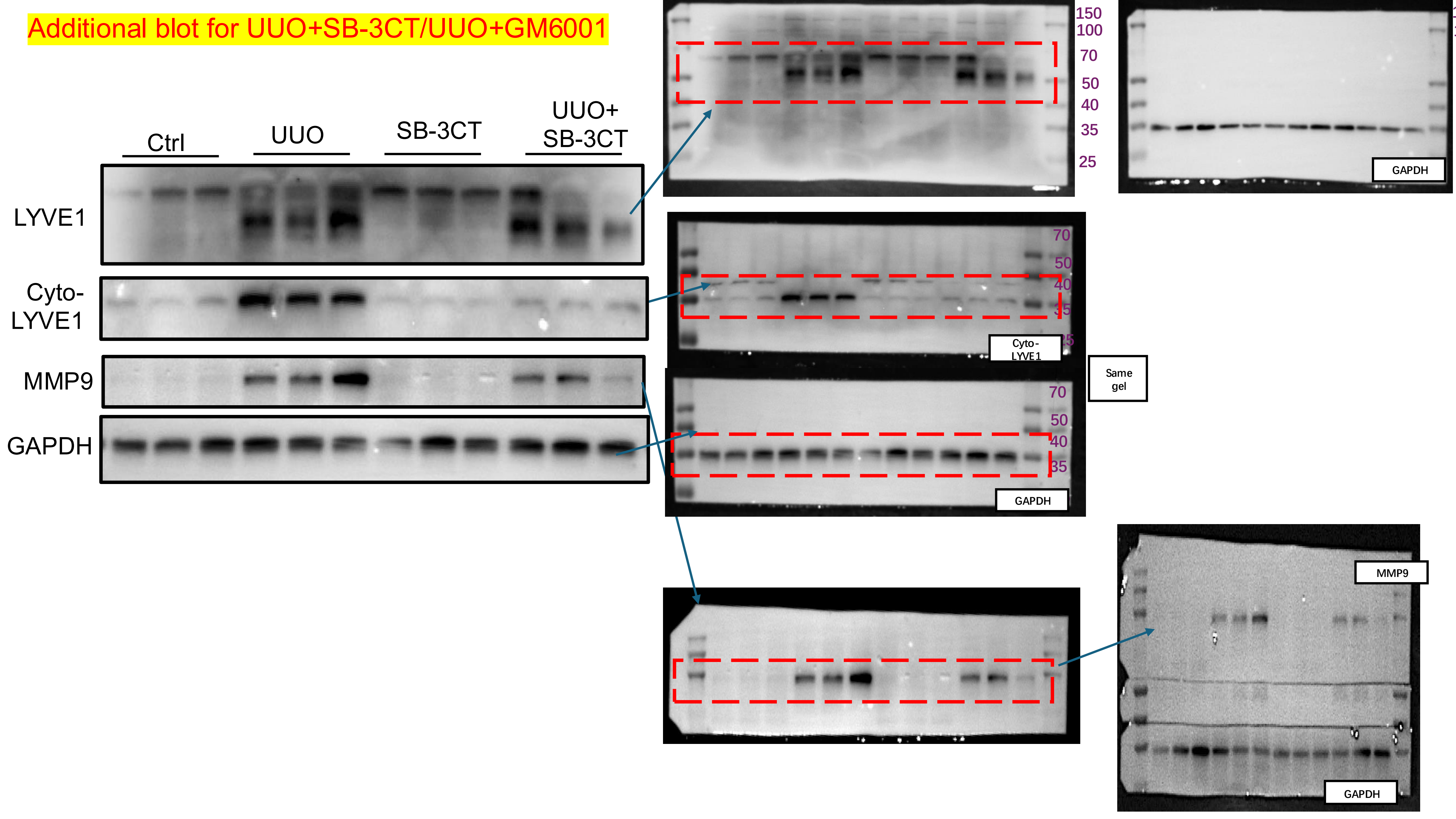

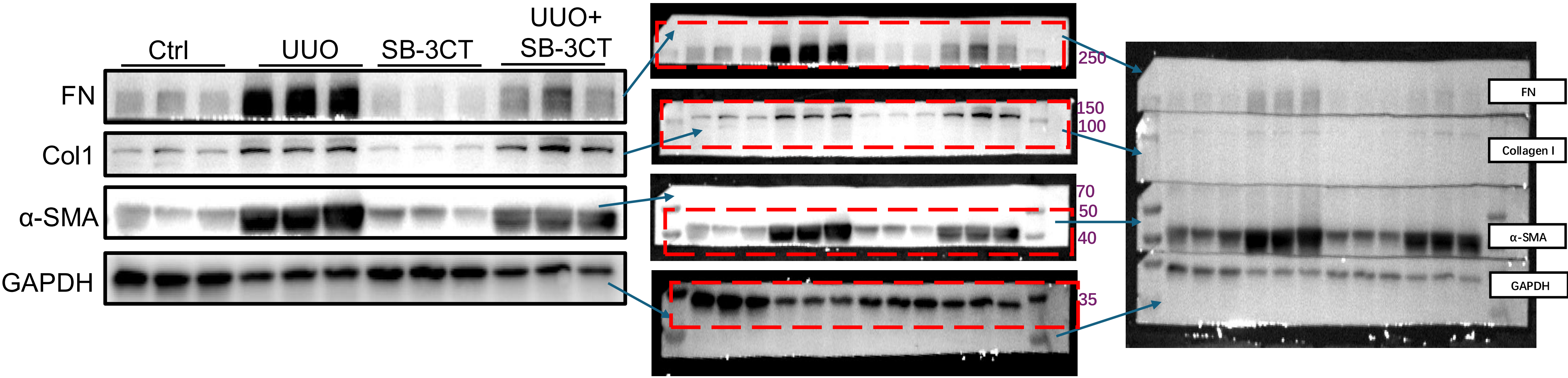
Ctrl
UUO
SB-3CT
UUO+ SB-3CT
FN
Col1
α-SMA
GAPDH
250
150
100
70
50
40
35
FN
Collagen I
α-SMA
GAPDH

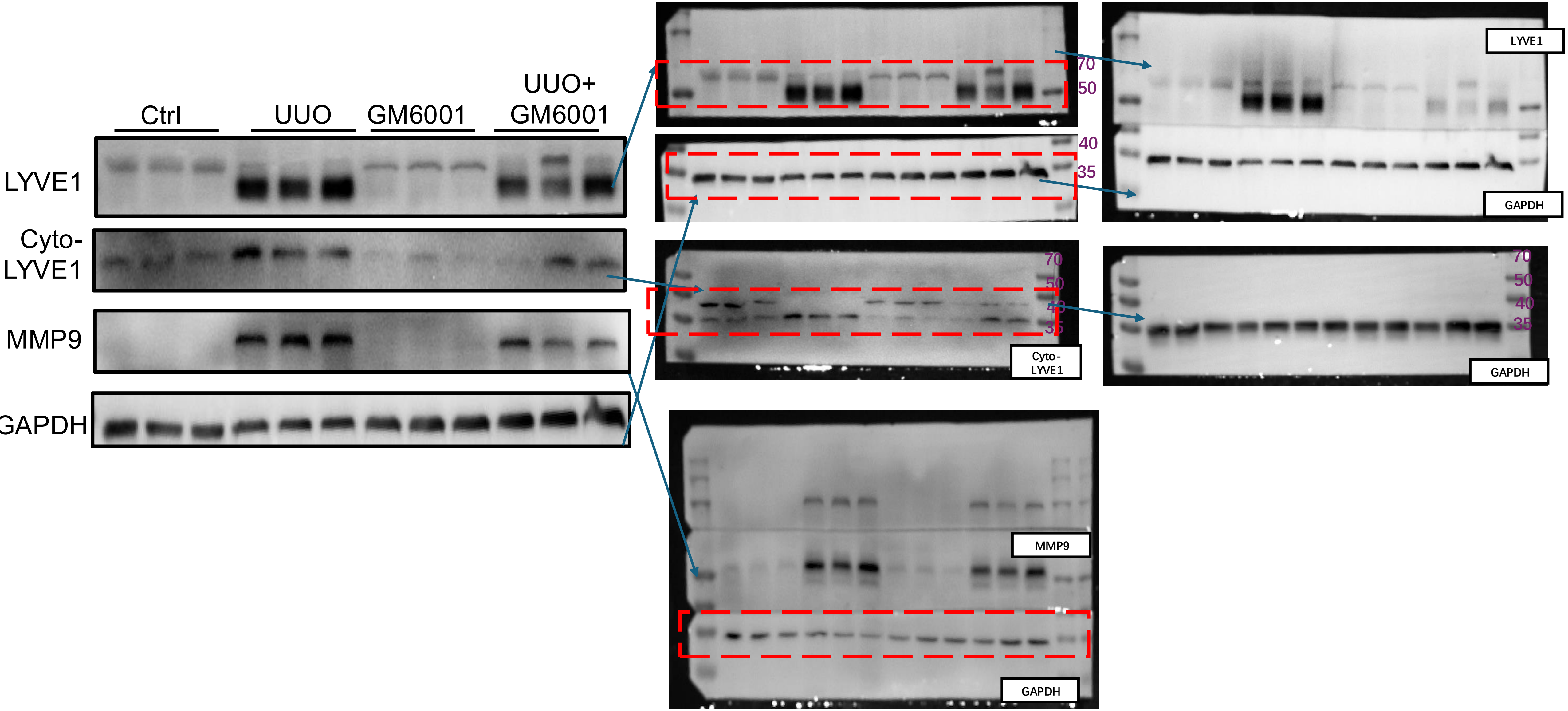
Ctrl
UUO
GM6001
UUO+ GM6001
LYVE1
Cyto-LYVE1
MMP9
GAPDH
70
50
40
35
LYVE1
GAPDH
70
50
40
35
Cyto-LYVE1
70
50
40
35
GAPDH
MMP9
GAPDH

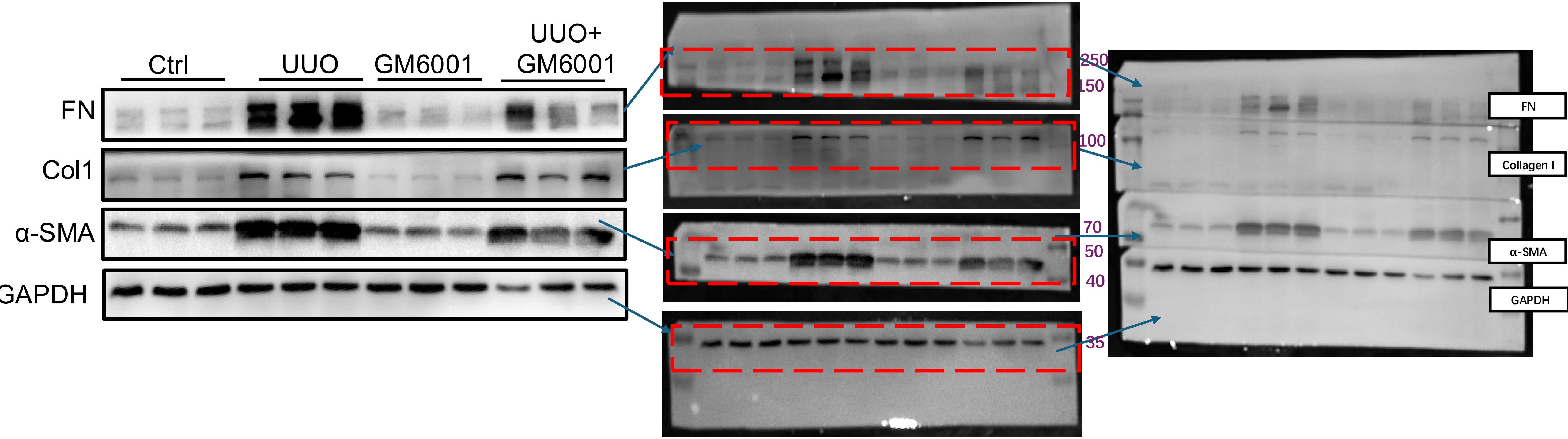

Ctrl
UUO
GM6001
UUO+
GM6001
FN
Col1
α-SMA
GAPDH
250
150
100
70
50
40
35
FN
Collagen I
α-SMA
GAPDH

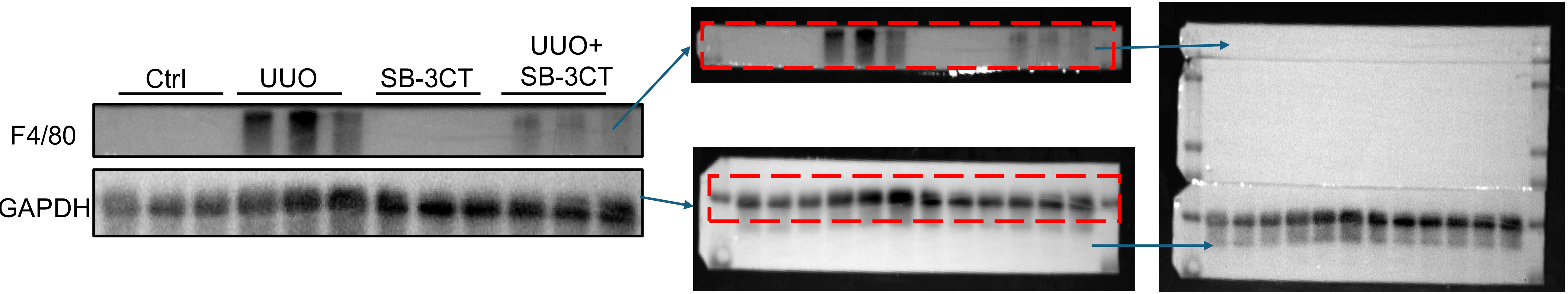
Ctrl
UUO
SB-3CT
UUO+
SB-3CT
F4/80
GAPDH

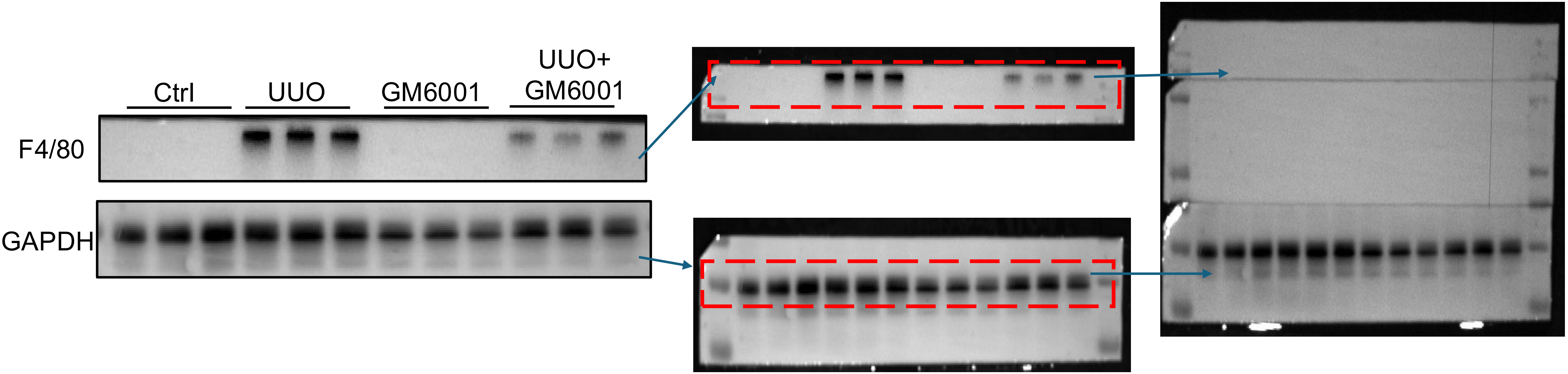
Ctrl
UUO
GM6001
UUO+
GM6001
F4/80
GAPDH
